# *mus-52* disruption and metabolic regulation in *Neurospora crassa*: Transcriptional responses to extracellular phosphate availability

**DOI:** 10.1371/journal.pone.0195871

**Published:** 2018-04-18

**Authors:** Maíra P. Martins, Eriston V. Gomes, Pablo R. Sanches, Wellington R. Pedersoli, Nilce M. Martinez-Rossi, Antonio Rossi

**Affiliations:** Department of Genetics, Ribeirão Preto Medical School, São Paulo University, Ribeirão Preto, São Paulo, Brazil; University of California Riverside, UNITED STATES

## Abstract

Advances in the understanding of molecular systems depend on specific tools like the disruption of genes to produce strains with the desired characteristics. The disruption of any mutagen sensitive (*mus*) genes in the model fungus *Neurospora crassa*, i.e. *mus-51*, *mus-52*, or *mus-53*, orthologous to the human genes *KU70*, *KU80*, and *LIG4*, respectively, provides efficient tools for gene targeting. Accordingly, we used RNA-sequencing and reverse transcription-quantitative polymerase chain reaction amplification techniques to evaluate the effects of *mus-52* deletion in *N*. *crassa* gene transcriptional modulation, and thus, infer its influence regarding metabolic response to extracellular availability of inorganic phosphate (Pi). Notably, the absence of MUS-52 affected the transcription of a vast number of genes, highlighting the expression of those coding for transcription factors, kinases, circadian clocks, oxi-reduction balance, and membrane- and nucleolus-related proteins. These findings may provide insights toward the KU molecular mechanisms, which have been related to telomere maintenance, apoptosis, DNA replication, and gene transcription regulation, as well as associated human conditions including immune system disorders, cancer, and aging.

## Introduction

Changes in the environment, including nutrient availability, can be sensed by all living organisms. Thus, living systems react to changes with appropriate cellular responses, such as regulation of growth, proliferation, metabolism, and apoptosis. The fungal lifestyle can be determined depending on the strategies for nutrient acquisition [1–3]. The uptake and utilization of nutrients including inorganic phosphate (Pi) is crucial in physiological metabolisms, such as energy transduction, maintenance of genetic information, cell growth, synthesis of membrane phospholipids, and cellular signalling processes in general [4]. The genetic and molecular mechanisms controlling the response to Pi starvation in *N*. *crassa* include at least five regulatory genes (*nuc-2*, *mak-2*, *preg*, *pgov*, and *nuc-1*). The extracellular Pi shortage is sensed by the NUC-2 protein, which transmits the signals downstream through a MAPK cascade, inhibiting the function of the PREG-PGOV complex, probably by a putative interaction with MAK-2. In turn, this allows translocation of the transcription factor NUC-1 into the nucleus, enabling the transcription of nucleases, phosphatases, phosphodiesterase (s), polyphosphate kinase, endopolyphosphatase, heat shock proteins, and Pi transporters [4–10].

Systematic gene disruption constitutes an important strategy for determining the functions of specific genes. In addition to the completion of *N*. *crassa* genome sequence, the availability of a set of mutant strains individually knocked-out in particular genes, such as the *mutagen sensitive* (*mus*) genes, provides an efficient tool for gene targeting [11–13]. In particular, *N*. *crassa mus-51*, *mus-52*, and *mus-53*, homologous to human *KU70*, *KU80*, and *LIG4* respectively, play a vital role in DNA repair, binding tightly to the DNA ends and direct their ligation independent of DNA homology [14]. The *ku* genes are also described as being associated with telomere maintenance, apoptosis processes, DNA replication, gene transcription regulation, and especially in non-homologous end-joining (NHEJ) of double-stranded DNA breaks [14,15]. In the absence of any of *mus* genes, the rate of homologous recombination (HR) is considerably increased, which markedly enhances the ease of conducting procedures for genetic alterations of targeted genes [11,13,16]. Otherwise, little information is available regarding transcriptional regulation in these kinds of genetically modified organisms [11,17].

In a recent report, the deletion of *N*. *crassa mus-52* gene was shown to affect the expression of genes related to holocellulolytic degradation and transcription factors that regulate these groups of enzymes [18]. In fact, the biological consequences of *mus-52* deletion are not yet well understood and sometimes even neglected. Thus, we hypothesize that the absence of MUS-52 causes profound changes in fungal physiology that affect the dynamics and regulation of diverse metabolic pathways; e.g., cell signalling, circadian rhythms, and others.

Aiming to contribute to increase the knowledge of the functionality of the gene mus-52 we utilized RNA-sequencing (RNA-Seq) and reverse transcription-quantitative polymerase chain reaction (RT-qPCR) techniques to identify transcriptional changes in two *Neurospora* strains (wild-type FGSC 2489 and mutant FGSC 9568), cultivated in medium containing low- and high-Pi concentrations. Our results indicated that the extracellular Pi availability influenced the expression of genes involved in several biological functions. The main affected gene groups were those associated with integral components of the membrane, such as transport, regulation, and cell signalling pathways, as well as genes involved in the nucleolus and protein synthesis. The absence of MUS-52 affected global gene transcription in all conditions tested, highlighting the expression of some specific gene groups, such as transcription factors (TFs), kinase proteins, circadian clock-controlled genes, oxi-reduction balance, phosphate pathways, and general metabolism.

To avoid the possible effects of *mus* mutations, as shown here, the group involved in the *Neurospora* knockout Project decided to cross the primary transformants to wild type to isolate homokaryotic knockout mutants free of these mutations [11]. Therefore, all homokaryons available at the Fungal Genetics Stock Center (http://www.fgsc.net/) are *mus*+. However, to some fungal species whose mechanism of crossing is not known the present results are relevant to alert about the consequence of the absence of MUS-52 to fungal physiology. These aspects must be taken into consideration if a double mutant is analysed.

## Results

For a comprehensive analysis of the *N*. *crassa* global transcriptome considering two biological variables, one environmental (phosphate availability) and the other genetic (*mus-52* gene disruption), we performed high-throughput sequencing (RNA-Seq) using the wild-type FGSC 2489 and mutant FGSC 9568 (Δ*mus-52*) strains cultivated in medium containing low- and high-Pi concentrations (see the Methods section). Thus, by aligning the mapped reads against the reference genome (ftp://ftp.broadinstitute.org/pub/annotation/fungi/neurospora_crassa/assembly/NC12/), it was possible to identify approximately 88% of the 10,082 protein-coding genes previously predicted in the *N*. *crassa* genome [19].

### Molecular analysis reveals condition-specific expression of several *N*. *crassa* genes

**Fig 1:**
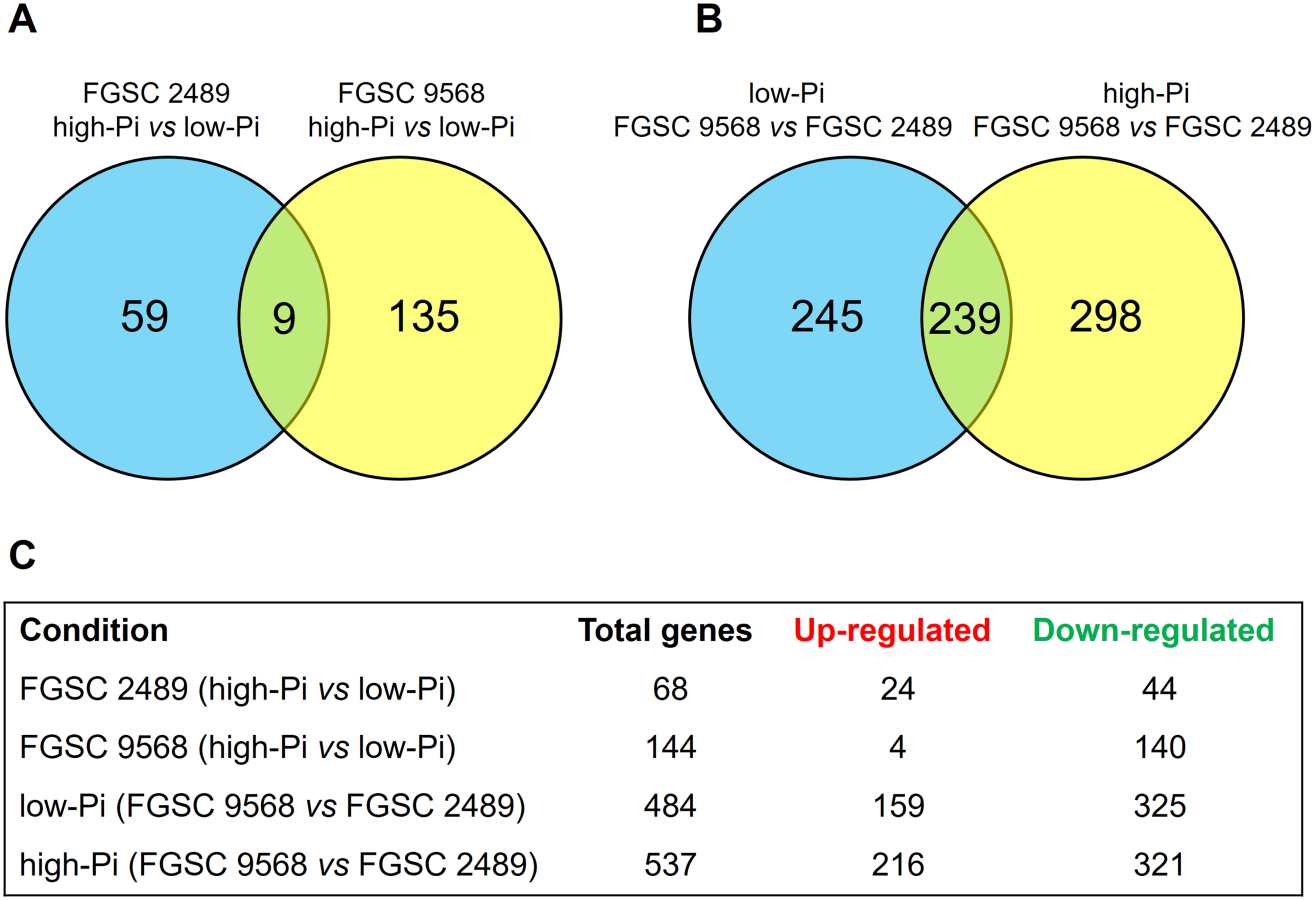
Overall expression analysis of *N*. *crassa* wild-type FGSC 2489 and mutant FGSC 9568 strains grown in medium containing different Pi availability (10 mM-high-Pi and 10 μM- low-Pi). (A) Venn diagram indicating the number of DEGs in the challenge condition (High-Pi) compared to the control condition (Low-Pi) in the wild-type FGSC 2489 strain (blue) and mutant FGSC 9568 (yellow). (B) Venn diagram indicating the number of DEGs by the mutant strain FGSC 9568 (test) compared to the control (FGSC 2489 strain) in medium containing low-Pi (blue) and high-Pi (yellow). (C) The table indicates the number of DEGs in both *N*. *crassa* strains and both media conditions. Thresholds for calling DEGs were (P ≤ 0.05) and up regulated → log2 (Fold Change) ≥ 1.5 / Down-regulated → log2 (Fold Change) ≤ −1.5.

Using the experimental setup described in the Material and Methods section with an adjusted *P*-value ≤ 0.05 and an independent filter [20], we identified a set of differentially expressed genes (DEGs) (S1 Table). Considering Pi availability as the first variable, and applying 2.8-fold change (that is, log2 fold change ≥ 1.5 or ≤−1.5) as the expression threshold, we have identified 68 DEGs, comprising 24 up- and 44 down-regulated genes (Fig 1A and 1C) in the wild-type FGSC 2489 strain. Among the most up-regulated genes affected by the change in Pi availability in this strain, five were categorized as integral components of membrane (NCU08447, NCU12021, NCU03921, NCU09173, and NCU06613).

By way of contrast, for the mutant FGSC 9568 strain cultured in the same Pi availability condition we have identified 144 DEGs, comprising 4 up- and 140 down-regulated genes; from these, 2 up- (NCU11307 and NCU00552) and 4 down-regulated (NCU03921, NCU04148, NCU09627, NCU00878) genes were highlighted as also related to integral membrane components (Table B in S2 Table). Moreover, nine of the identified DEGs were shared by both strains. It was also identified that 59 exclusive DEGs existed in the wild-type FGSC 2489 and 135 existed in the mutant FGSC 9568 strains (Fig 1A and 1C).

In both Pi availability culture conditions, it was possible to observe how deeply the disruption in *mus-52* affected the transcriptional modulation in the *N*. *crassa* mutant strain. The fold change expression value between the wild-type FGSC 2489 and the mutant FGSC 9568 identified 484 DEGs in the low-Pi condition, wherein 159 genes were up-regulated, 325 were down-regulated, and 245 were expressed exclusively under low-Pi conditions (Fig 1B and 1C). The gene NCU04276 showed the highest down-regulation observed throughout this study, being approximately 970-fold reduced (Log2 = −9.92; Table C in S2 Table). In addition, this gene presented the same strong down-regulation expression pattern in the high-Pi condition as well (Log2 = −8.87; Table D in S2 Table). NCU04276 encodes a protein with 99 amino acids, having 96% and 85% identity to hypothetical proteins from *Neurospora tetrasperma* and *Sordaria macrospora*, respectively, and 100% identity with a hypothetical protein from *Pseudomonas syringae*. In addition to possible phosphorylation and N-myristoylation sites, the *in silico* scanning of the protein sequence using the PROSITE tool (http://prosite.expasy.org/prosite.html), a database of protein domains and functional prediction, indicated the presence of a C-terminal microbody targeting signal (Gly, Arg, and Ile) as the last three amino acids.

In high-Pi concentration culture medium, 537 DEGs were identified, of which 216 were up- and 321 down-regulated, with 298 genes expressed exclusively in this culture condition. Furthermore, 239 DEGs were shared by both *N*. *crassa* strains in both culture conditions (Fig 1B and 1C). The multidrug resistant protein coding gene (NCU00754) also showed a strong down-regulation (675-fold [log2 = −9.4] and 265-fold [log2 = −8.05]) in both low- and high-Pi conditions, respectively (Tables C and D in S2 Table), which is notable because its transcription regulation is also highly dependent on MUS-52.

Relative expressions of the 23 mutagen-sensitive (*mus*) genes were investigated in both strains in all tested conditions. Disruption of mutagen sensitive 52 (*mus-52*) affected two other *mus* genes in *N*. *crassa*. *mus-53* was the most affected among the *mus* genes, being up-regulated approximately 5-fold (log2 = 2.30) in the low-Pi condition. *mus-26* was up-regulated approximately 4.5-fold (log2 = 2.14) in the mutant strain, mainly in the high-Pi condition, although it also presented a relative up-regulation of approximately 3.2-fold (log2 = 1.67) in low-Pi as well (S3 Table). The *mus-11* gene is adjacent to the most down-regulated NCU04276 DEG. Considering that they are on opposite strands of the DNA locus and in a different orientation, *mus-11* did not present differential expression in any of the conditions investigated. Thus, they probably do not share the same regulatory system.

**Fig 2:**
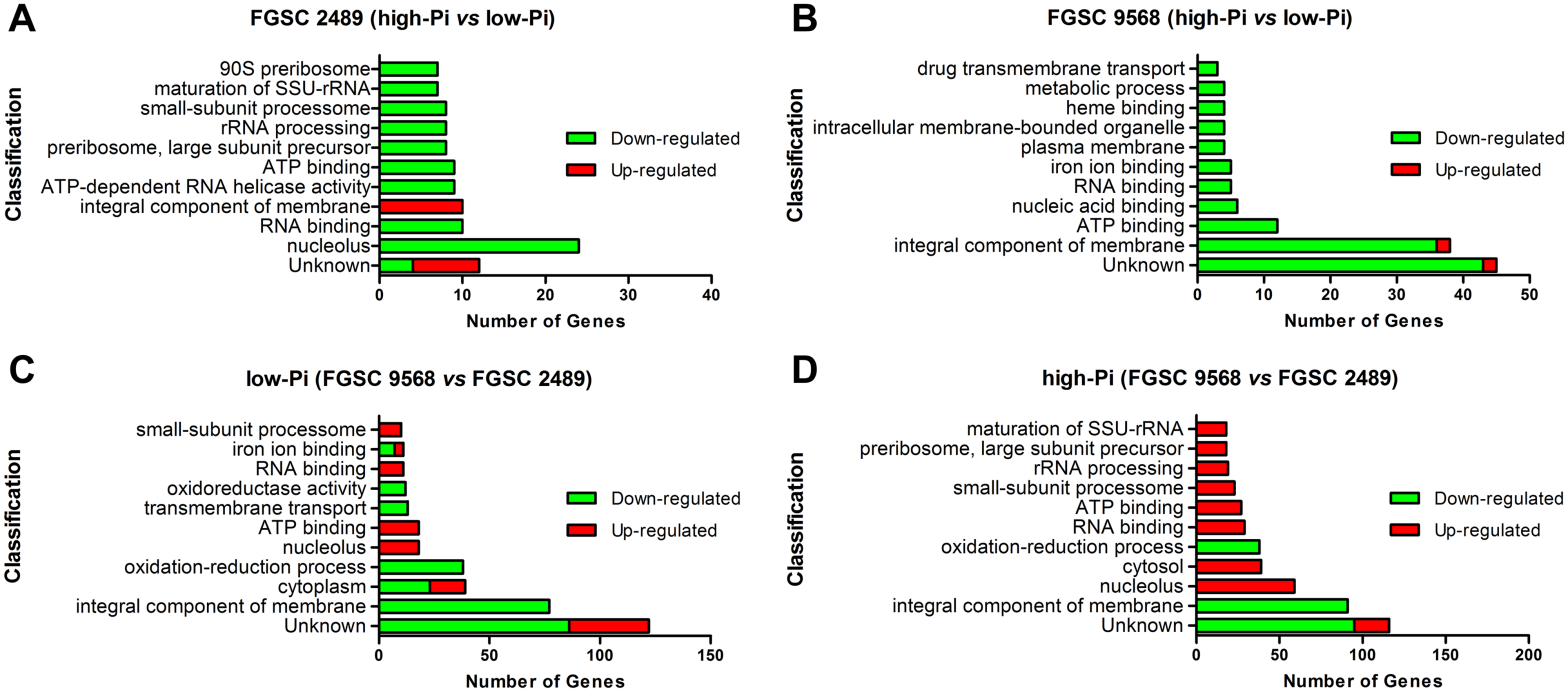
Gene Ontology (GO) enrichment analysis. (A) The 10 classes of genes in the *N*. *crassa* FGSC 2489 strain most affected by Pi availability. (B) The 10 classes of genes in the *N*. *crassa* FGSC 9568 strain most impacted by Pi availability. (C, D) The 10 classes of the most affected genes in *N*. *crassa* FGSC 9568 with respect to the wild-type strain in the low-Pi (C) and high-Pi (D) culture condition. Significantly enriched categories (P ≤ 0.05) are shown.

Gene Ontology (GO) enrichment analysis indicated that high-Pi availability down-regulates the majority of DEGs in the wild-type FGSC 2489 strain compared with the low-Pi condition, highlighting the 24 nucleolus-related and other genes that, directly or indirectly, are involved in the process of protein synthesis. The only class of up-regulated genes showed ten genes associated with an integral component of the membrane (Fig 2A). Additionally, eight up- and four down-regulated genes were categorized as having unknown function. Notably, for the mutant FGSC 9568 strain, the high-Pi condition down-regulated all the classes categorized, except for genes with unknown function of which 43 were down- and two up-regulated (Fig 2B). Moreover, considering the integral component of the membrane group, the change of Pi availability induced a down-regulation of 36 genes and up-regulation of only two (NCU00552 and NCU11307) in the mutant strain, although these genes differed from those described for the wild-type FGSC 2489 strain in the same gene group (Fig 2A and 2B). One of the most important points to consider is that the absence of MUS-52 affected the modulation of several genes classified as integral membrane components regardless of Pi availability, down-regulating 77 genes in low-Pi and 91 in high-Pi conditions (Fig 2C and 2D), along with another group specifically classified as transmembrane transport with 13 down-regulated genes (Fig 2C). It was also observed that deletion of *mus-52* affected other classes of genes, such as down-regulating genes of the oxidation-reduction process (38 genes in both low-Pi and high-Pi), while up-regulating genes related to the nucleolus (18 and 59 genes in low- and high-Pi, respectively) and the ATP-binding gene group (18 and 27 genes in low- and high-Pi, respectively). Furthermore, the *mus-52* mutation also affected several genes with unknown function, with 86 genes up- and 36 down-regulated under the low-Pi condition, and 21 and 95 genes up- and down-regulated, respectively, under high-Pi conditions (Fig 2C and 2D).

### TF modulation

**Fig 3:**
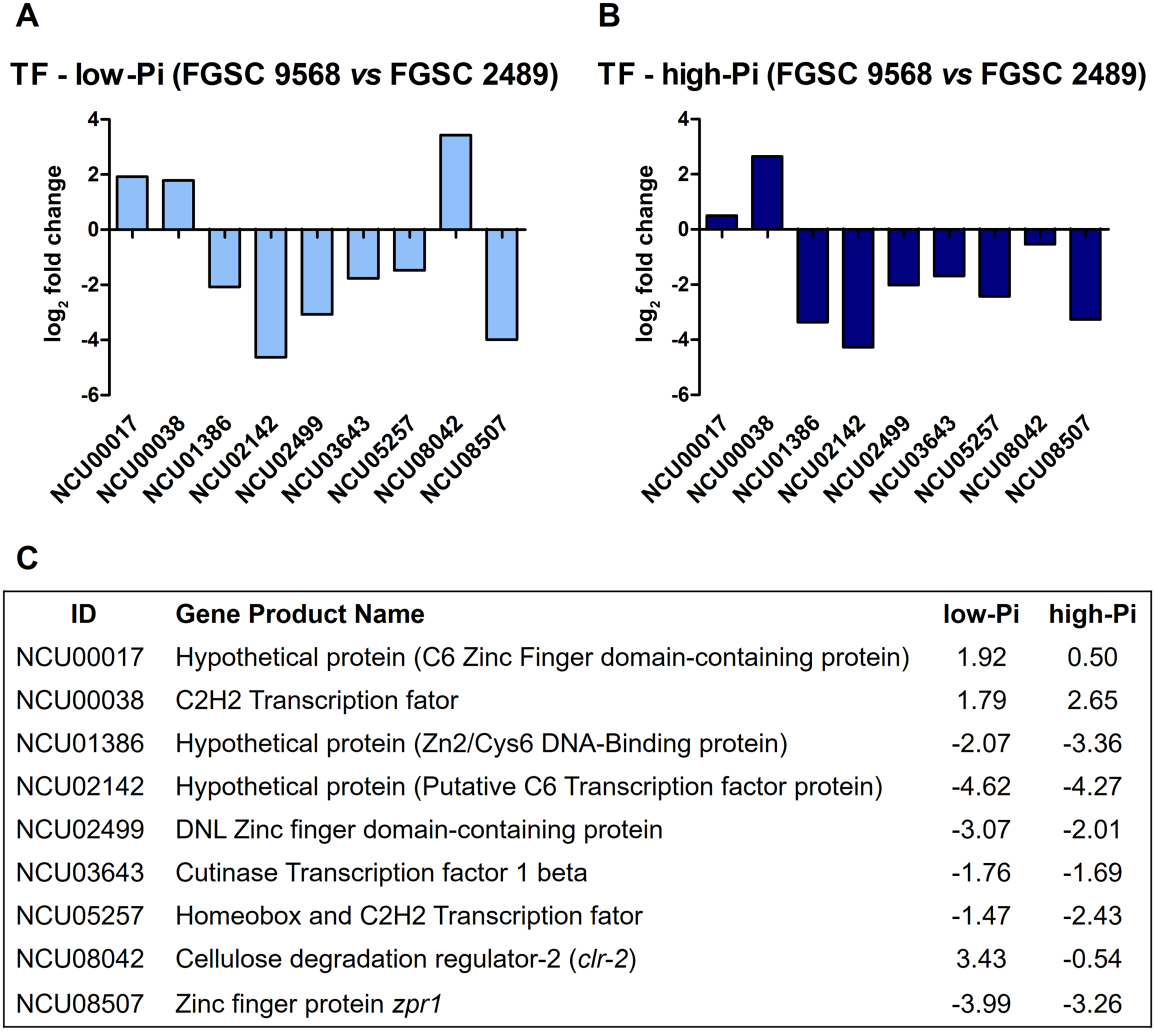
Expression analysis of the *N*. *crassa* genes encoding TFs in wild-type FGSC 2489 and mutant FGSC 9568 strains grown in media with different Pi availability. (A, B) Fold change expression ratio of 9 genes encoding TFs in mutant FGSC 9568 *vs* FGSC 2489 grown in medium containing low-Pi (A) or high-Pi (B) availability. (C) Gene ID and description of the TFs analysed and their respective relative expression (log2) under conditions of low- and high-Pi availability.

The change expression values of 9 specific and randomly selected differentially expressed TF genes between mutant FGSC 9568 *vs* wild-type FGSC 2489 strains were analysed by the RNA-Seq and validated by RT-qPCR techniques, to illustrate the effects of Pi availability and deletion of the *mus-52* gene in the modulation of these kinds of regulators. In low-Pi condition, six TF genes were down-regulated, highlighting the putative C6 transcription factor protein (NCU02142) coding gene, which was also down-regulated in the high-Pi condition. Although the NCU05257 gene displayed a fold change value (log2 = −1.47) lower than our expression threshold, it was nevertheless considered in our analysis. Conversely, four genes were up-regulated in low-Pi condition, highlighting *cellulose degradation regulator-2* (*clr-2*, NCU08042), which was transcribed approximately ten-fold higher (log2 = 3.42) in the mutant FGSC 9568 compared with the wild-type FGSC 2489 strain (Fig 3A and 3C). Under the high-Pi condition, only one TF gene was up-regulated (coding for a C2H2 transcription factor, NCU00038), according to our expression threshold, and six TF genes were down-regulated, highlighting a putative C6 transcription factor (NCU02142); these comprised the most affected TF coding genes in this Pi condition (Fig 3B and 3C).

### Kinase gene expression profile

**Fig 4:**
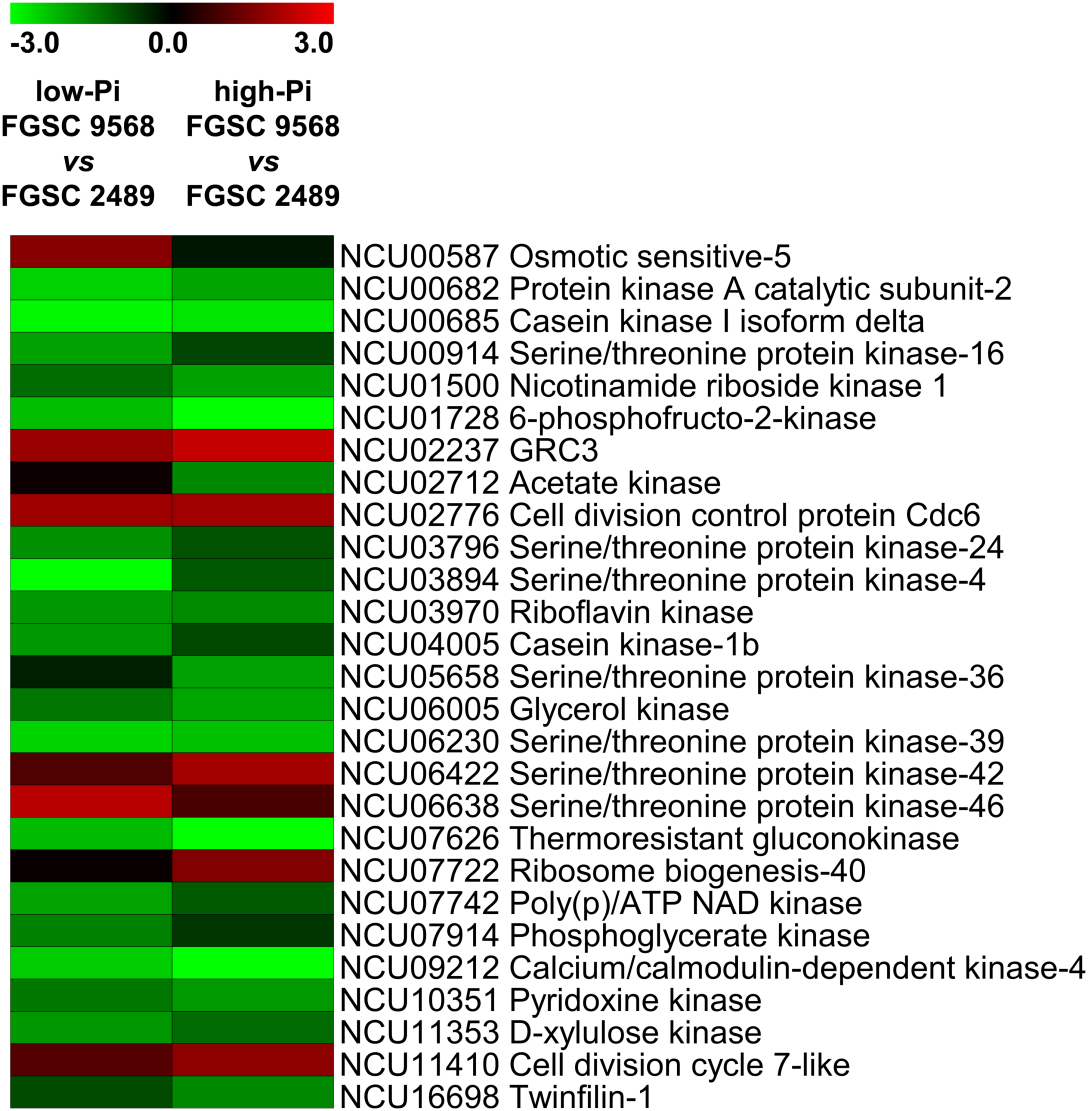
Kinase and kinase-related gene expression profile (heat map) of *N*. *crassa* FGSC 9568 mutant *vs* wild-type strains, grown in low- and high-Pi availability conditions. Expression scale is represented as log2-fold change.

The change expression value of the 27 kinase and kinase-related genes between the mutant FGSC 9568 *vs* wild-type FGSC 2489 strains were used to construct an expression profile (heat map). The large serine-threonine (S/T) class of the eukaryotic protein kinase superfamily stands out among these kinase genes as having several genes down-regulated. Serine/threonine protein kinase-4 (NCU03894) was the most affected kinase gene in the low-Pi condition (log2 fold change = −4.64). In high-Pi condition, the most down-regulated DEG was the thermoresistant gluconokinase (NCU07626; log2 fold change = −4.48), (Fig 4 and S4 Table). Another specific down-regulated kinase was CK-1a (NCU00685), which is part of the oscillator core of *N*. *crassa* clock-controlled genes (ccgs) (fold change log2 = −2.93 and −2.71 in low- and high-Pi conditions respectively), (Fig 4 and S4 Table).

### Circadian clock-controlled genes

**Fig 5:**
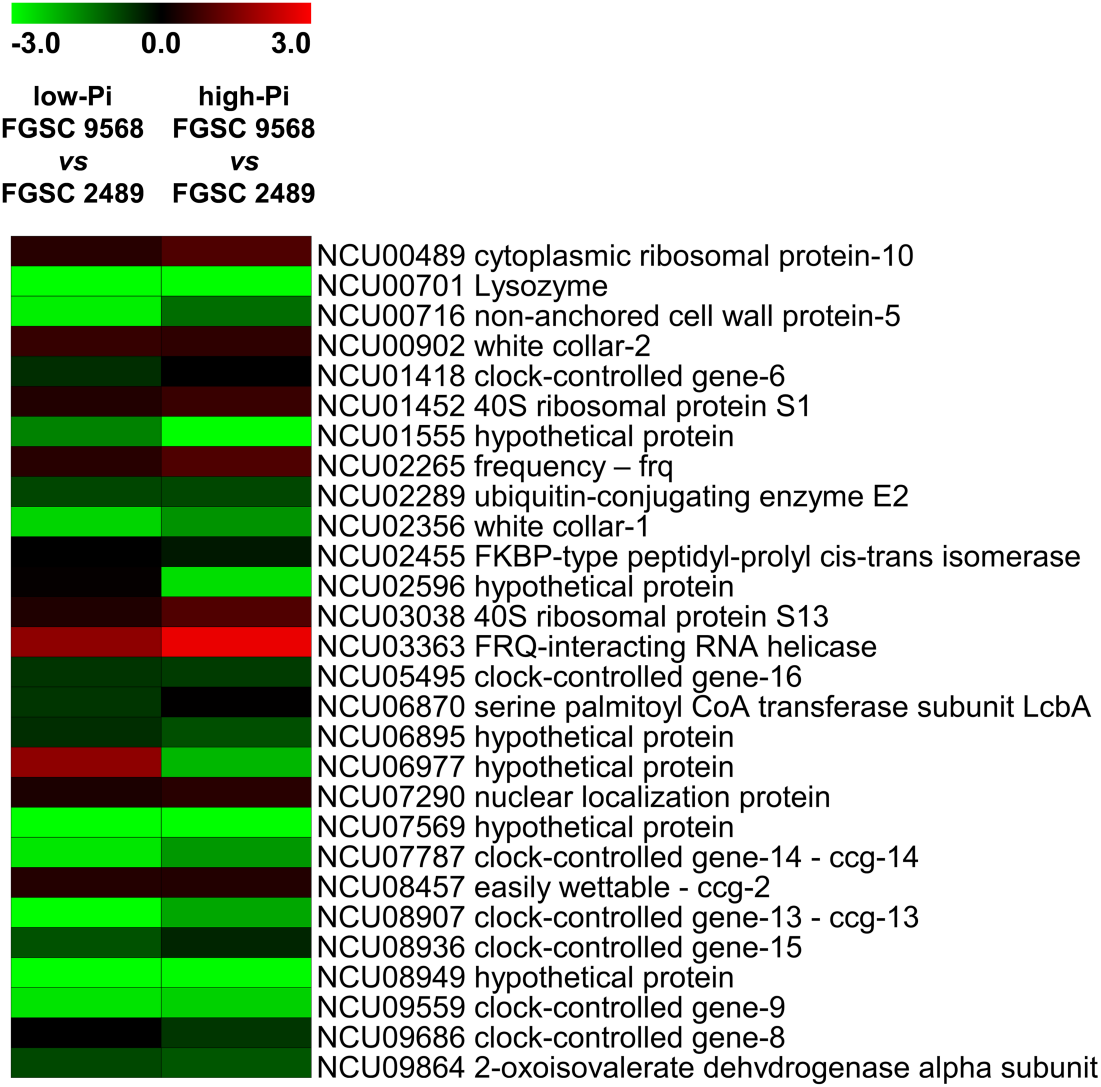
Circadian clock-controlled gene expression profile (heat map) of *N*. *crassa* FGSC 9568 mutant *vs* wild-type strains, grown in low- and high-Pi availability conditions. Expression scale is represented as log2-fold change.

We next analysed the fold change expression profile of the 28 circadian clock-controlled genes. Among the five genes (*frq*, *wc-1*, *wc-2*, *frh*, and *ck-1a*) of the core oscillator in *N*. *crassa*, *wc-1* (NCU02356) and the previously cited *ck-1a* (NCU00685) were down-regulated in the mutant FGSC 9568 compared with the wild-type FGSC 2489 strain. Conversely, *frq* (NCU02265) and *wc-2* (NCU00902) were slightly up-regulated but were not considered as DEGs. The gene for FRQ-interacting RNA helicase (*FRH*, NCU03363) was strongly up-regulated. Furthermore, the majority of the other ccgs were down-regulated, with *lysozyme* (*lyz*, NCU00701) highlighted as the most down-regulated gene identified among the ccgs, being decreased approximately 105-fold (log2 = −6.70) in low-Pi and approximately 125-fold (log2 = −6.98) in high-Pi conditions (Fig 5 and S5 Table).

### General metabolism-related genes

**Fig 6:**
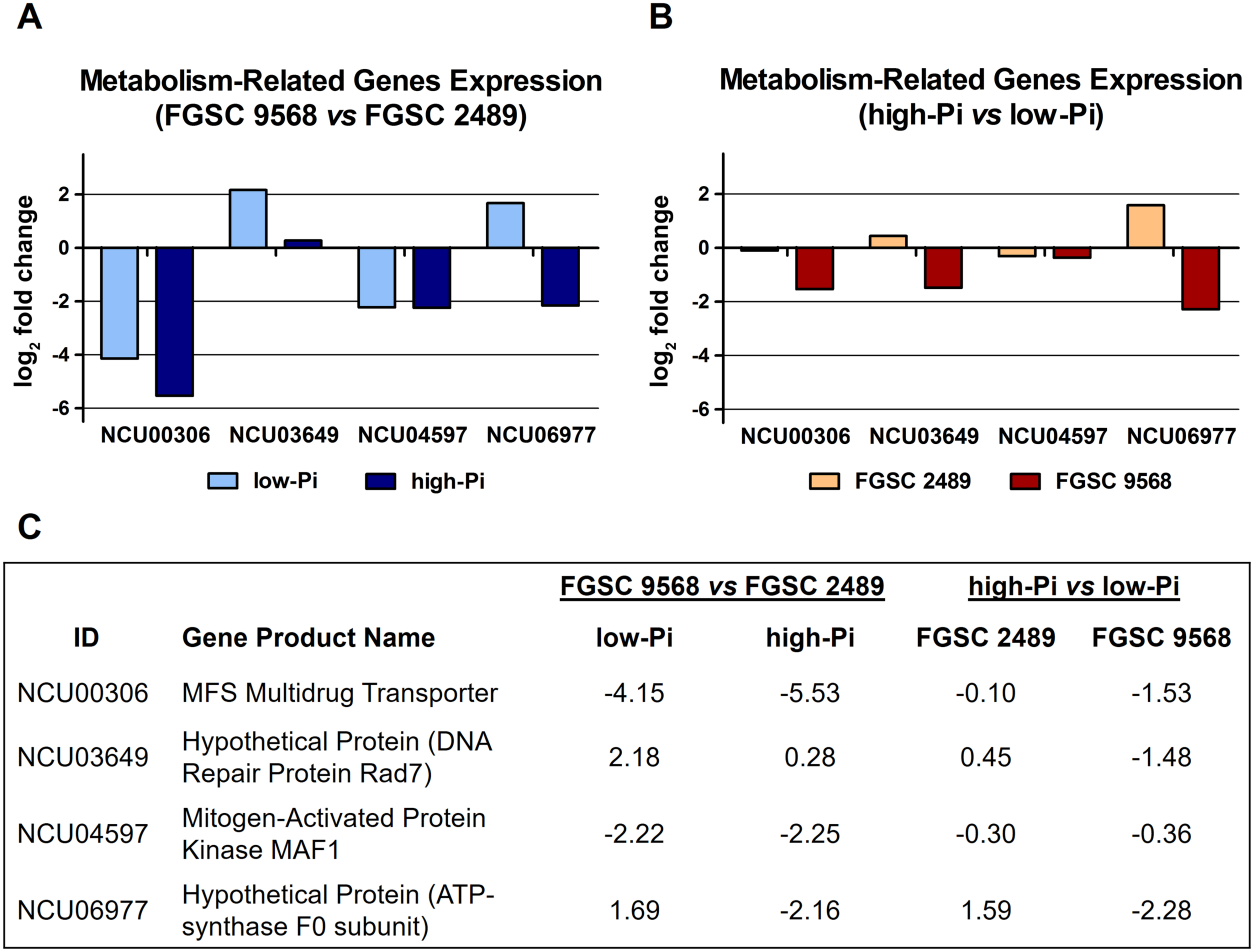
Expression analysis of the genes related to metabolism in *N*. *crassa* wild-type FGSC 2489 and mutant FGSC 9568 grown in media with different Pi availability. (A) Fold change expression ratio of 4 genes related to the metabolism of the mutant FGSC 9568 *vs* FGSC 2489 strains cultured in medium containing low-Pi (light blue columns) and high-Pi (dark blue columns). (B) Fold change expression ratio of 4 genes related to the metabolism in high-Pi *vs* low-Pi growth condition of both wild-type FGSC 2489 (orange columns) and mutant FGSC 9568 (brown columns) strains. (C) Description of the metabolism-related genes analysed in the *N*. *crassa* wild-type and mutant FGSC 9568 strains and their respective relative expression (log2) under conditions of low- and high-Pi availability.

The relative expression of four general metabolism-related genes and the fold change expression value between the mutant FGSC 9568 *vs* wild-type FGSC 2489 strains in both low- and high-Pi culture conditions were also analysed. We found that two genes (NCU00306 and NCU04597) were affected by the *mus-52* gene disruption, with both being down-regulated, regardless of the availability of Pi (in low- and high-Pi; Fig 6A and 6C), highlighting the MSF multidrug transporter coding gene (NCU00306) as being markedly down-regulated. Only the gene that codes for a hypothetical protein (NCU03649), which when blasted against NCBI GenBank showed approximately 70% identity with the *Metarhizium acridum* (CQMa 102) DNA repair protein Rad7, was up-regulated solely in the Pi starvation condition. Notably, the *N*. *crassa* metabolism-related hypothetical protein NCU06977, when blasted against the NCBI database, showed > 70% identity with a *Colletotrichum graminicola* ATP synthase, F0 subunit. The fold change expression value of NCU06977 in the wild-type FGSC 2489 strain under high-Pi *vs* low-Pi conditions indicated a strong up-regulation. Conversely, the fold change expression value of the mutant FGSC 9568 cultured in the same condition indicated an equivalent down-regulation (Fig 6B and 6C). In comparison, no significant difference was observed in the relative expression profile of two putative DNA methyltransferase (DMT) genes (*dim-2* [NCU02247] and *rid-1* [NCU02034]), reported as critical for the genome repeat-induced point mutation (RIP) defence system in *N*. *crassa* [21], which suggests that the absence of MUS-52 does not interfere with RIP in *N*. *crassa*.

### Gene expression validation

Expression analysis by qRT-PCR of 13 selected genes using independent RNA samples validated the RNA-Seq data. The results of both experiments were compared with respect to the log2 ratio between *mus-52* mutant and wild-type FGSC 2489 strains (S6 Table). The correlation between the Illumina RNA-Seq and qPCR results, obtained from biological replicates, was strong and was statistically significant (Pearson correlation, r = 0.97, *P* < 0.001).

## Discussion

The acquisition of Pi by microorganisms depends on ambient signals, which are detected by cellular sensors that initiate the flow of intracellular information. The transcriptional activation of Pi-related genes triggers an intricate mechanism for Pi homeostasis including phosphatases to release inorganic Pi from organic molecules and polyphosphates, and membrane phosphate permeases to import the Pi into the cells [12,22]. Several studies have utilised the ascomycete fungus *N*. *crassa* for insights regarding the molecular mechanism that controls the response to phosphate availability. A suppression subtractive hybridization approach has been applied to identify genes modulated by the transcription factor NUC-1 [6]. This approach has identified *hex-1*, a gene classified as septal pore-associated (an organelle specific to filamentous ascomycete fungi, known as the Woronin body, which acts in the prevention of cytoplasmic bleeding after hyphal injury) as well as its association with extracellular Pi and pH changes [6]. The same approach was used to identify transcripts differently expressed in the *N*. *crassa* strain lacking the phosphate sensor NUC-2, which regulates several genes associated with the phosphorus-sensing network, such as transport, transcriptional regulation, signal transduction, metabolism, protein synthesis, and development [5,23,24]. The authors highlighted the *mak-2* gene, which codes for a mitogen-activated protein kinase (MAPK-MAK-2). In a subsequent work, using a *N*. *crassa* mutant (Δ*mak-2*) strain grown under phosphate-shortage conditions, the authors reported evidence that, in addition to involvement in metabolic routes (e.g., the isoprenylation pathway), *mak-2* participates in the hierarchical phosphate-signalling pathway, being the fifth element in the control system of the Pi shortage response [4,5,25,26].

In the current study, we utilised high-throughput genomic approaches to investigate *N*. *crassa* transcriptional regulation under different phosphate availability conditions. We observed that in the *N*. *crassa* wild-type FGSC 2449 strain, the nucleolus-related genes were the most down-regulated DEGs in high-Pi *vs* low-Pi (Fig 2A). Notably, the nucleolus participates in tRNA precursor processing, biogenesis and processing of rRNA, gene silencing, senescence, stress sensing (influencing p53 protein activities), cell cycle regulation, tumour suppression, assembly of signal recognition particles (machinery responsible for the proper localization of nascent proteins to their specific cellular localization), and modulation of telomerase functions [27–31].

Another example of a phosphate-repressive gene observed in the wild-type FGSC 2489 strain is that coding for *cyanide hydratase* (NCU04697, Table A in S2 Table), an enzyme involved in the hydrolysis of cyanide to formamide [32]. In fungi, cyanide is produced as a mechanism to provide a nitrogen and carbon source in fruiting bodies, and under damage or stress conditions [33].

The gene group categorized by the GO database as an ‘integral component of membrane’ is also notable. It is the only group up-regulated (except those with unknown functions) by phosphate availability in the wild-type FGSC 2489 strain (Fig 2A). This group is characterized by gene products and protein complexes having at least some part of their peptide sequence embedded in the hydrophobic region of the membrane. Although the majority of affected genes from this specific group were classified as hypothetical proteins, we highlight two distinct members. The mitochondrial chaperone bcs1 (NCU03921), which has a single transmembrane domain crossing the mitochondrial inner membrane, is reported to be involved in the respiratory chain Complex III (also known as Ubiquinol-cytochrome c oxidoreductase or cytochrome bc_1_ complex) assembly. This complex is responsible for actively pumping protons across the inner membrane from the mitochondrial matrix to the intermembrane space, thereby creating a membrane potential. The protons are thus attracted back to the matrix, passing through ATP synthase (complex V), whereby the influx of protons generates the energy necessary for the conversion of ADP to ATP [34–36]. Accordingly, it is reasonable to suggest that the observed up-regulation of this gene (Table A in S2 Table), owing to the condition of high availability of phosphate, stimulated the mitochondrial activity and consequently increased the ATP synthesis in the *N*. *crassa* wild-type FGSC 2489 strain. This hypothesis is supported by the differentially expressed level shown by a putative ATP synthase-F0 subunit (NCU06977). In high-Pi condition, the wild-type FGSC 2489 strain exhibits up-regulation of this gene (Fig 6B and 6C). This result is consistent with both increased Pi availability and ATP production by oxidative phosphorylation in mitochondria. In contrast, such increase in the transcription level is not observed in the mutant FGSC 9568 strain. The fold change expression value observed in the mutant compared to the wild-type FGSC 2489 strain shows a converse response to the variations of Pi availability, with the highest transcription level occurring in low-Pi and the lowest in high-Pi (Fig 6A and 6C).

The other up-regulated member of the ‘integral component of membrane’ category, modulated in high Pi condition, is an ammonium transporter (NCU06613). This gene encodes a protein present in the major kingdoms of living organisms, which displays an AmtB conserved domain that is associated with the transportation of inorganic compounds (Table A in S2 Table). Similar to phosphate, ammonium is an important component of the metabolic process for cell growth and development, being the main nitrogen source in all organisms. Thus, ammonium transporter proteins such as ammonia channel protein AmtB comprise important structures not only for transport but also to sense the environmental ammonium availability [37].

The previously identified *mus* genes are sensitivity to a wide range of physical and chemical mutagens (e.g., UV, X-Ray, methyl methanesulfonate, and histidine) [38–41]. Some of these *mus*-mutations have been used for specific gene disruption and recombinant DNA integration investigations. These tools, which require HR at the target locus, can be considered as a first step in the rapid functional analyses of genes, such as those involved in virulence of pathogenic organisms [42]. Disruption of *N*. *crassa* genes involved with the NHEJ process (i.e., *mus-51*, *mus-52*, and *mus-53*) dramatically increases the frequency of HR. Evidence suggests that the NHEJ system drives the random integration of exogenous DNA into ectopic sites of the genome, whereas HR promotes targeted site integration, thus representing an invaluable genetic resource to facilitate physiologic, molecular, and biochemical studies [11,13,16,42].

In the current study, we used the *N*. *crassa* FGSC 9568 mutant strain to analyse the effect of the absence of MUS-52 protein on global gene regulation under conditions of different Pi availability. Notably, the regulation of the overall expression in the mutant strain relative to the availability of Pi when compared to wild-type FGSC 2489 alignment was markedly distinct. In general, the data suggest that considerable effort and consequently higher energy consumption was required by the mutant strain (considering the number of DEGs in both Pi conditions) for its adaptation to a variable environment when compared with the wild-type FGSC 2489 strain (Fig 1). In fact, the data indicate that several metabolic pathways of the mutant FGSC 9568 follow alternative routes or even opposite expression pattern of their respective genes. For example, the nucleolus-related group, as categorized by the GO database (previously described for the wild-type FGSC 2489 strain), showed that all DEGs in the mutant strain were up-regulated in both Pi conditions, highlighting the high-Pi with 59 up-regulated genes (Fig 2C and 2D). Among other aspects, the data suggest an increase in the protein synthesis process, which is likely required for mutant adaptation to the changed environmental conditions of phosphate availability. In contrast, the ‘integral component of membrane’ group consisted of >70 down-regulated genes in the mutant compared with the wild-type FGSC 2489 strain in both Pi conditions, with the multidrug resistance coding genes (NCU00306 and NCU00754) being highlighted. Although further analyses are required, these data indicate that the *N*. *crassa* mutant strain may be more sensitive to some chemical compounds, which could be of interest for those studying other organisms with *mus*-52 (or its equivalents) disrupted. By comparing the expression pattern of ‘integral component of membrane’ group genes in the wild-type FGSC 2489 strain, we observed that only ten genes were up-regulated in high-Pi, whereas 36 genes were down-regulated in the mutant strain in the same experimental condition (Fig 2A and 2B). Thus, cellular functions such as transport across membranes (considering both the plasma membrane and cell organelle membranes) as well as cell signalling from membrane receptors may have been affected by the absence of MUS-52. This hypothesis is supported by the relative expression level of other specific membrane-related groups, such as ‘drug transmembrane transport’ and ‘plasma membrane proteins’ (Fig 2B). Moreover, approximately 50 DEGs associated with the oxidation-reduction process in mutant FGSC 9568 were down-regulated in both, low- and high-Pi conditions compared with the wild-type FGSC 2489 strain (Fig 2C and 2D). This difference in expression may result in consequences for cellular metabolism, especially with regard to the intracellular oxi-reduction balance; i.e., considering that several fungal activities, such as host colonization by the fungal pathogens, depend on the efficiency of the ROS detoxification systems, which must scavenge ROS, maintain reduced redox states at the microenvironments, and repair ROS-triggered cellular and membrane damage [43].

After changes to the environment, such as nutritional availability, the signal transduction pathways will likely be linked with the expression of targeted genes by specific TFs. The transcription, or lack thereof, of a particular TF gene may represent a total metabolism modification and indicate transcriptional rewiring of the regulatory mechanism [44]. Therefore, in the current study, the expression analysis of 9 randomly selected TFs presented an informative expression pattern upon the comparison between *N*. *crassa* wild-type FGSC 2489 and mutant FGSC 9568 strains. Among these, seven genes (NCU00038; NCU001386; NCU02142; NCU02499; NCU03643; NCU05257, and NCU08507) were concomitantly modulated in both phosphate availability conditions, with NCU00038 as the only single DEG up-regulated in both Pi conditions (Fig 3). Most of the TFs analysed exhibited a zinc finger domain (a short peptide with the secondary structure stabilized by a zinc ion bound with conserved cysteine and histidine residues) that could be classified into several groups according to their diverse zinc-binding motifs [45,46]. Notably, NCU00038, which contains Cys2His2 (C2H2, a classical zinc finger [CZF] motif) and encodes a protein that binds both DNA and RNA molecules [47] was the only gene up-regulated in both Pi conditions. Thus, considering the previously discussed up-regulation of the nucleolus-related genes (Fig 2C and 2D), which also occurred in both Pi conditions, we could speculate that the up-regulation of this specific TF might indicate a possible increase of protein synthesis, as it can be required for the initiation of 5S RNA synthesis by RNA polymerase III, as described for TFIIA [47]. Moreover, the NCU00038 expression is also reported in other contexts, such as growth, conidiation, metabolism, sexual reproduction, virulence, drug resistance, and stress response [46,48]. Conversely, the marked down-regulation of the other six TFs, in both Pi conditions (Fig 3), may lead to an unpredictable transcription regulation of genes in the whole genome. Alternatively, the identification of *clr-2* (NCU08042), which constitutes a highly conserved fungal-specific zinc binuclear cluster transcription factor that is responsible for the induction of genes encoding enzymes associated with cellulose deconstruction, as the most up-regulated TF (Fig 3A) might indicate that the *N*. *crassa* mutant FGSC 9568 strain would be a better producer of cellulose degradation enzymes than the wild-type FGSC 2489 strain in low-Pi conditions. However, further analyses are necessary regarding this issue.

Kinase proteins comprise another relevant group in the regulation of metabolic pathways and, directly or indirectly, gene expression processes. These enzymes display a central role in signalling pathways and, thus, in regulating proliferation, survival, apoptosis, general metabolism, transcription, and differentiation [49,50]. In the present study, we show that absence of MUS-52 down-regulates the majority of the kinase proteins in both low- and high-Pi conditions. This expression pattern may indicate an interference arising from the *mus-52* disruption rather than the Pi availability in the transcriptional response of the protein kinases, especially of a specific group known as serine/threonine kinases (STKs) (Fig 4 and S4 Table). STKs can be further classified into groups based on their catalytic domains, with over a hundred members associated with several cellular functions predicted in *N*. *crassa* [19,51].

The phosphorylation process is also associated with the regulation of the circadian clocks, which synchronize the physiological and cellular activities of an organism for the appropriate times of day, a process that is well described in *N*. *crassa*. Phosphorylation of the frequency clock protein (FRQ), which is one of the main proteins related to the regulation of the circadian clock, is a key step in ‘time counting’ and consequently in specific gene expression for a giving period of the day [52]. In the present study, the down regulation of CK-1a (NCU00685) observed in the mutant FGSC 9568 strain compared with the wild-type FGSC 2489 (Fig 4 and S4 Table) may indicate that the circadian metabolism control system is affected by the absence of MUS-52 in *N*. *crassa*. In this case, it might affect the FRQ phosphorylation pattern, leading to a delay in its degradation and, consequently, in the circadian clock itself. A down-regulation of CK-1b (NCU04005), a homologue to CK-1a, was also observed (Fig 4 and S4 Table); this protein can phosphorylate FRQ *in vitro* as well, although it was described having no essential function in the *N*. *crassa* circadian clock [53,54]. These findings indicate further that the *mus-52* disruption affects the expression of several ccgs and, consequently, the cellular processes such as stress response, intermediary metabolism, protein synthesis, and development [55].

Furthermore, considering that the down-regulation of CK-1a might lead to hypophosphorylation of FRQ, and consequently to a delay on its degradation, it would be reasonable to hypothesize that *wc-1* and *wc-2* would be up-regulated, and thus, the *frq* gene would be increased as well as a result of WC-1 and WC-2 dimerization and consequent formation of the WCC. In practice, a discrete up-regulation of *frq* and *wc-2* were indeed observed (S5 Table). A strong down-regulation of *wc-1* was also noted, which might suggest that the FRQ phosphorylation pattern interferes directly with the transcription of *wc-1*, or even that there is another factor, dependent on CK-1a, acting on the regulation of this gene, which does not interfere in *wc-2* and *frq* transcription. In addition, the vast majority of the other ccgs were down-regulated in the mutant FGSC 9568 transcriptome compared to the wild-type FGSC 2489 strain, corroborating the hypothesis that the temporal pattern of the mutant is altered and may lead to unpredictable physiological consequences.

Notably, the two most affected genes (NCU04276 and NCU05498) observed in the *mus-52* mutant strain upon culture in both high- and low-Pi, encode for hypothetical proteins (Tables C and D in S2 Table). The NCU05498 gene sequence did not show homology with other genes coding for proteins with known biological function. In comparison, the NCU04276 gene sequence shares high degree of identity between *N*. *tetrasperma*, *S*. *macrospora*, and *P*. *syringae*, and is probably associated with microbodies (i.e., glyoxysomes and peroxisomes) owing to the tripeptide targeting signal at the C-terminal region. In phytopathogenic fungi (e.g., *Sclerotinia sclerotiorum* and *Sclerotium rolfsii*) the microbodies contribute to virulence. These fungi produce and secrete oxalic acid that, during the infection process, acts in the degradation of the plant cell wall. Two key enzymes in the production of oxalate (isocitrate lyase and glyoxylate dehydrogenase) are located in the peroxisomes [56]. In human disorders, microbodies are related to peroxisomal dysfunction, such as cerebrohepatorenal (Zellweger) syndrome, Refsum disease, adrenoleukodystrophy, and acatalasaemia [57]. Thus, down-regulation of this gene may affect the functioning of these organelles in *N*. *crassa*.

Up-regulation of the mutagen sensitive genes *mus-53* and *mus-26* (S3 Table) as a consequence of *mus-52* disruption, is not surprising because both are described as being part of the DNA repair system. In *N*. *crassa*, *mus-53* encodes a DNA ligase (homologous to human *Lig4*) that belongs to the NHEJ recombination repair system, which can also act in a MUS-52-independent pathway [16]. In addition to being related to UV mutagenesis, *mus*-26, which is homologous to yeast REV7, is also a DNA damage-inducible gene [58].

We have shown here that the absence of MUS-52 profoundly alters several metabolic pathways that are extremely important for physiological homeostasis, which involve protein functions such as cell signalling, phosphorylation, redox balance, oxidative stress response, gene transcription, and molecular adjustments to circadian rhythm via ccgs.

A plausible explanation for these abnormal metabolic changes may, at present, only be given speculatively. It is difficult to attest whether MUS-52 affects all of these cellular processes by direct interaction or, likely in an indirect manner, reducing the access of enhancers and TFs to the promoter region of some specific genes. Conversely, these adjustments could act by affecting one or several of these pathways thus triggering a type of cascade effect. Therefore, this effect could elicit transcriptional feedback loops of specific genes from different pathways (mainly negative transcriptional feedback, as shown here for the majority of the gene groups from the mutant strain), creating molecular links as a sort of crosstalk. Such links may exist between alternative signalling cascades driven by kinases and membrane proteins, and gene transcription regulation driven by TFs and related transcriptional proteins. Accordingly, organisms containing these derived mutations deserve further and profound molecular evaluation.

## Materials and methods

### *N*. *crassa* strains and culture conditions

*N*. *crassa* St.L.74-OR23-1VA (wild-type FGSC 2489; control strain) and the FGSC 9568 strain (FGSC No. 9568; *mat a*, *mus-52*::*hyg*) were from the Fungal Genetics Stock Center, FGSC, Kansas State University, Manhattan, KS, USA (www.fgsc.net). These strains were maintained on solid Vogel’s minimal medium, pH 5.8 containing 2% sucrose at 30°C [4]. Gene knockout in the mutant strain was confirmed by qRT-PCR using specific oligonucleotides by comparison to the amplification of genomic DNA from the wild-type FGSC 2489 strain.

Conidia from 6 day-cultures of each *N*. *crassa* strain (approximately 10^6^ cells ml^−1^) were germinated for 5 h at 30°C in an orbital shaker (200 rpm), in low- and high-Pi media (final concentrations, 10 μM or 10 mM Pi, respectively). Media were supplemented with 44 mM sucrose as the carbon source, and the pH was adjusted to 5.4 (buffered with 50 mM sodium citrate), as previously described [25,59]. The mycelium was harvested on Millipore filters (pore size 0.22 μm), rinsed 3 times with 5–10 ml aliquots of distilled water, frozen in liquid nitrogen, and stored at −80°C until use.

### RNA extraction

Frozen mycelia from both *N*. *crassa* strains were used for isolation of total RNA through a TRIzol RNA kit (Invitrogen Life Technologies, Carlsbad, CA, USA) and treated with RNase-free DNAse I (Thermo Fisher, Waltham, MA, USA). RNA concentrations were determined using a NanoDrop ND-1000 spectrophotometer (Wilmington, DE, USA), and RNA integrity was verified using both agarose electrophoresis and the Agilent Bioanalyser platform 2100 (Agilent Technologies, Santa Clara, CA, USA).

### RNA-Seq analyses

A total of four cDNA libraries were sequenced (*N*. *crassa* wild-type FGSC 2489 and FGSC 9568 mutant strains, cultivated in high- and low-Pi concentration media), with their respective biological triplicates corresponding to the paired libraries, using an Illumina HiSeq2000 sequencer, to generate 100 bp paired-end reads. *FastQC* software was used to visualize the library quality before and after trimming. For quality and -sequence filtering, a *Phred score* lower than 20 was employed to remove sequencing bases from the read ends. Filtered reads were mapped onto the *N*. *crassa* genome (ftp://ftp.broadinstitute.org/pub/annotation/fungi/neurospora_crassa/assembly/NC12/) using *Bowtie2* software [60]. The mean coverage and alignments of the transcripts were visually inspected using *Integrative Genomics Viewer* software (IGV) [61,62]. After the library alignment and quality filter steps, the reads count values were obtained and used to calculate the expression variation of the transcripts from different conditions, considering the statistical significance of the differential gene expression [63]. In addition, an independent filter was applied to increase the accuracy of the data, to minimize the occurrence of false positives owing to low read counts [20]. Afterward, the False Discovery Rate statistical test was applied on the significance of the gene expression value among the samples, using the *DESeq* package, manipulated in the R statistical environment [63]. After that, an exclusion criterion of 2.8-fold (that is, log2 fold change ≥ 1.5 or ≤−1.5) was applied as an expression threshold. Functional annotation of the DEGs was performed according to *GO* [64], using *Bast2GO* software [65]. GO term enrichment was performed using the *BayGO* algorithm [66]. To identify metabolic pathways in which genes could be modulated in response to phosphate availability, annotation was performed using *Kegg Orthology* [67], using the *Kegg Automatic Annotation Server* (KAAS) and *N*. *crassa* as a reference organism. Expression graphics were constructed using GraphPad Prism v 5.1 Software (LaJolla, CA, USA).

### Idiomorph-related genes

As the *N*. *crassa* wild-type FGSC 2489 and the mutant FGSC 9568 strains have different mating systems, conferred by the idiomorphic *mat* locus (*mat A* and *mat a*, respectively), we used the microarray data available in the GEO database (GSE41484) [68] to exclude the DEGs that could be regulated by the mating type system during the asexual development of *N*. *crassa* (S7 Table). In addition, we also used a highly conserved MAT1-1-1 DNA binding motif (5′-CTATTGAG-3′) [69], which shares similarities with known DNA-binding motifs of proteins involved in the regulation of sexual reproduction in yeast and embryonic development in vertebrates, to analyse 1000 bp upstream of the entire *N*. *crassa* genome and exclude the DEGs that contain this DNA binding motif in their respective promoter regions (S8 Table).

### RT-qPCR analyses

The expression profile of selected genes was quantified by performing qRT-PCR using Power SYBR Green PCR Master Mix (Applied Biosystems, Foster City, CA, USA) with the StepOnePlus Real-Time PCR System (Applied Biosystems). One μg of total RNA, extracted from mycelia, was reverse transcribed into cDNA using the High Capacity cDNA Reverse Transcription Kit (Applied Biosystems) according to the manufacturer’s instructions. The PCR protocol included an initial denaturation step at 50°C for 2 min and at 95°C for 10 min, followed by 40 cycles of 95°C for 15 s and 60°C for 1 min. A dissociation curve was generated at the end of each PCR cycle to verify the amplification of a single product. Each reaction was done in triplicate. The *actin* (NCU04173) and *β-tubulin* (NCU04054) genes were used as endogenous controls. Relative expression levels of each gene were calculated using the Livak (2^−ΔΔCt^) method [70]. Statistical analyses were performed using a two-tailed Student’s *t*-test.

## Supporting information

S1 Table Genes of *N*. *crassa* modulated in response to *mus-52* deletion (FGSC 9568 *vs* FGSC 2489). (DOCX)

S2 Table Top 10 most significantly up- or down-regulated differentially expressed genes. (DOCX)

S3 Table Log2 fold-change mutagen sensitive gene expression. (DOCX)

S4 Table Log2 fold-change kinase gene expression. (DOCX)

S5 Table Log2 fold-change circadian clock-related gene expression. (DOCX)

S6 Table Comparison of the gene expression levels assayed by RNA-Seq and qPCR approaches. (DOCX)

S7 Table Genes potentially involved in the mating-type mechanism. (DOCX)

S8 Table *In silico* prediction of the DNA-binding motif for the regulator MAT1-1-1 (5’-CTATTGAG-3’). (DOCX)
