# Supplementary material for: mus-52 disruption and metabolic regulation in Neurospora crassa: Transcriptional responses to extracellular phosphate availability
Source: PLoS One. 2018 Apr 18;13(4):e0195871. doi: 10.1371/journal.pone.0195871 (PMC5905970; doi:10.1371/journal.pone.0195871)
Supplement: S1 Table — (DOCX) [file pone.0195871.s001.docx]

**S1 Table - Genes of *N. crassa* modulated in response to *mus-52* deletion (FGSC 9568 *vs* FGSC 2489)**

**S1A Table –Genes only modulated in low-Pi**

| **ID** | **low-Pi** | **high-Pi** | **Gene Product Name** |
| --- | --- | --- | --- |
| NCU08640 | 3.99 |  | hypothetical protein |
| NCU08042 | 3.43 |  | cellulose degradation regulator-2 |
| NCU00802 | 3.39 |  | hypothetical protein |
| NCU05001 | 2.98 |  | cycloheximide-inducible-1 |
| NCU04778 | 2.88 |  | carbonic anhydrase |
| NCU10009 | 2.87 |  | ATP-binding cassette transporter |
| NCU04058 | 2.77 |  | hypothetical protein |
| NCU02086 | 2.75 |  | hypothetical protein |
| NCU10077 | 2.71 |  | hypothetical protein |
| NCU08852 | 2.66 |  | poly(ADP-ribose) polymerase |
| NCU07553 | 2.65 |  | hypothetical protein |
| NCU07325 | 2.64 |  | conidiation-10 |
| NCU08821 | 2.63 |  | hypothetical protein |
| NCU10006 | 2.61 |  | hypothetical protein |
| NCU09183 | 2.56 |  | kynureninase |
| NCU08603 | 2.54 |  | ankyrin repeat protein |
| NCU08760 | 2.53 |  | glycosylhydrolase family 61-5 |
| NCU10597 | 2.51 |  | hypothetical protein |
| NCU05230 | 2.46 |  | hypothetical protein |
| NCU05277 | 2.46 |  | hypothetical protein |
| NCU04260 | 2.42 |  | oxidoreductase domain-containing protein |
| NCU06264 | 2.30 |  | mutagen sensitive-53 |
| NCU02456 | 2.30 |  | potassium transporter |
| NCU00331 | 2.28 |  | hypothetical protein |
| NCU08816 | 2.27 |  | hypothetical protein |
| NCU03383 | 2.27 |  | hypothetical protein |
| NCU00878 | 2.27 |  | hypothetical protein |
| NCU09565 | 2.26 |  | hypothetical protein |
| NCU02512 | 2.25 |  | hypothetical protein |
| NCU02521 | 2.25 |  | hypothetical protein |
| NCU06946 | 2.25 |  | hypothetical protein |
| NCU09903 | 2.24 |  | elongation factor 3 |
| NCU09570 | 2.19 |  | glutathione transferase |
| NCU03649 | 2.18 |  | hypothetical protein |
| NCU01896 | 2.13 |  | hypothetical protein |
| NCU04931 | 2.12 |  | hypothetical protein |
| NCU03355 | 2.12 |  | calpain-5 |
| NCU03372 | 2.11 |  | nonspecific lipid-transfer protein |
| NCU04342 | 2.11 |  | hypothetical protein |
| NCU05555 | 2.11 |  | hypothetical protein |
| NCU05128 | 2.10 |  | hypothetical protein |
| NCU17062 | 2.10 |  | hypothetical protein |
| NCU09698 | 2.05 |  | hypothetical protein |
| NCU07159 | 2.04 |  | proteinase T |
| NCU04786 | 2.04 |  | SNF2 family helicase/ATPase |
| NCU02940 | 2.04 |  | hypothetical protein |
| NCU02049 | 2.03 |  | hypothetical protein |
| NCU05609 | 1.98 |  | hypothetical protein |
| NCU01081 | 1.98 |  | hypothetical protein |
| NCU07791 | 1.97 |  | hypothetical protein |
| NCU08733 | 1.96 |  | set-domain histone methyltransferase-9 |
| NCU04378 | 1.96 |  | DNA replication initiation factor Cdc45 |
| NCU03591 | 1.94 |  | ATP-dependent bile acid permease |
| NCU09151 | 1.93 |  | resistant to Spore killer |
| NCU00017 | 1.92 |  | hypothetical protein |
| NCU00261 | 1.91 |  | CTP synthase |
| NCU01629 | 1.89 |  | hypothetical protein |
| NCU00846 | 1.88 |  | hypothetical protein |
| NCU05393 | 1.88 |  | hypothetical protein |
| NCU02241 | 1.88 |  | DNA replication regulator sld-2 |
| NCU02776 | 1.87 |  | cell division control protein Cdc6 |
| NCU00711 | 1.85 |  | multidrug resistance protein fnx1 |
| NCU05210 | 1.84 |  | ultraviolet sensitive-2 |
| NCU07185 | 1.83 |  | hypothetical protein |
| NCU05784 | 1.83 |  | hypothetical protein |
| NCU01271 | 1.82 |  | spermidine-1 |
| NCU00835 | 1.81 |  | hypothetical protein |
| NCU00542 | 1.79 |  | DUF914 domain membrane protein |
| NCU00232 | 1.79 |  | hypothetical protein |
| NCU01457 | 1.78 |  | ubiquitin C-terminal hydrolase |
| NCU03650 | 1.77 |  | DNA repair protein RAD16 |
| NCU05194 | 1.77 |  | DNA replication licensing factor mcm6 |
| NCU09335 | 1.74 |  | hypothetical protein |
| NCU06130 | 1.74 |  | hypothetical protein |
| NCU08009 | 1.73 |  | DNA replication licensing factor mcm3 |
| NCU09566 | 1.73 |  | hypothetical protein |
| NCU05254 | 1.73 |  | ribose-phosphate pyrophosphokinase |
| NCU03569 | 1.72 |  | hypothetical protein |
| NCU07363 | 1.72 |  | hypothetical protein |
| NCU08660 | 1.71 |  | hypothetical protein |
| NCU06431 | 1.70 |  | 40S ribosomal protein S22 |
| NCU09010 | 1.70 |  | hypothetical protein |
| NCU06559 | 1.68 |  | 3-hydroxyisobutyrate dehydrogenase |
| NCU00821 | 1.67 |  | sugar transporter |
| NCU04963 | 1.66 |  | high-affinity glucose transporter |
| NCU08267 | 1.65 |  | activating signal cointegrator 1 complex subunit 3 |
| NCU07497 | 1.64 |  | hypothetical protein |
| NCU05897 | 1.64 |  | l-fucose permease |
| NCU04088 | -1.63 |  | hypothetical protein |
| NCU09798 | -1.65 |  | aryl-alcohol dehydrogenase |
| NCU05958 | -1.65 |  | hypothetical protein |
| NCU03755 | -1.68 |  | flavin dependent monooxygenase |
| NCU08048 | -1.69 |  | NAD dehydrogenase |
| NCU09343 | -1.69 |  | hypothetical protein |
| NCU00980 | -1.70 |  | hypothetical protein |
| NCU06717 | -1.70 |  | arsenical pump-driving ATPase |
| NCU03796 | -1.71 |  | serine/threonine protein kinase-24 |
| NCU06416 | -1.71 |  | thymine dioxygenase |
| NCU09600 | -1.72 |  | dienelactone hydrolase |
| NCU04720 | -1.72 |  | nitrate nonutilizer-6 |
| NCU04592 | -1.72 |  | 3-oxoacyl-(acyl-carrier-protein) reductase |
| NCU11201 | -1.74 |  | phosphoglycerate mutase |
| NCU11368 | -1.74 |  | 5',5'''-P-1,P-4-tetraphosphate phosphorylase 2 |
| NCU03888 | -1.74 |  | DUF500 and SH3 domain-containing protein |
| NCU02788 | -1.74 |  | ric8-like |
| NCU07875 | -1.74 |  | hypothetical protein |
| NCU06603 | -1.75 |  | ThiJ/PfpI family protein |
| NCU07456 | -1.75 |  | riboflavin-5 |
| NCU09735 | -1.75 |  | short-chain dehydrogenase |
| NCU03643 | -1.76 |  | cutinase transcription factor 1 beta |
| NCU04814 | -1.77 |  | DUF21 and CBS domain-containing protein |
| NCU09422 | -1.77 |  | hypothetical protein |
| NCU09345 | -1.77 |  | no message in thiamine-1 |
| NCU05771 | -1.77 |  | hypothetical protein |
| NCU01571 | -1.77 |  | hypothetical protein |
| NCU11215 | -1.78 |  | RING-10 protein |
| NCU11353 | -1.78 |  | D-xylulose kinase |
| NCU06862 | -1.78 |  | hypothetical protein |
| NCU10933 | -1.78 |  | hypothetical protein |
| NCU06319 | -1.79 |  | glycosyl hydrolase family 76-4 |
| NCU01850 | -1.79 |  | peroxin 16 |
| NCU00813 | -1.79 |  | disulfide isomerase |
| NCU08169 | -1.80 |  | hypothetical protein |
| NCU03970 | -1.80 |  | riboflavin kinase |
| NCU03303 | -1.80 |  | hypothetical protein |
| NCU17019 | -1.80 |  | hypothetical protein |
| NCU09160 | -1.81 |  | HPP family protein |
| NCU01747 | -1.82 |  | glycerophosphocholine phosphodiesterase Gde1 |
| NCU09395 | -1.82 |  | hypothetical protein |
| NCU07360 | -1.83 |  | DnaJ domain-containing protein |
| NCU05259 | -1.83 |  | acyl-CoA desaturase 1 |
| NCU01801 | -1.84 |  | hypothetical protein |
| NCU06462 | -1.84 |  | hypothetical protein |
| NCU09255 | -1.85 |  | acetate non-utilizing protein 9 |
| NCU03747 | -1.86 |  | hypothetical protein |
| NCU01911 | -1.86 |  | GDP/GTP exchange factor Sec2p |
| NCU06050 | -1.87 |  | hypothetical protein |
| NCU11307 | -1.88 |  | CCC1 |
| NCU02282 | -1.88 |  | aureobasidin-resistance protein |
| NCU01670 | -1.88 |  | type 1 phosphatase regulator ypi-1 |
| NCU07320 | -1.88 |  | phosphatidylinositol transporter |
| NCU00187 | -1.88 |  | menadione-induced gene-11 |
| NCU00847 | -1.90 |  | hypothetical protein |
| NCU04230 | -1.90 |  | acetate utilization-3 |
| NCU03678 | -1.92 |  | ssh-4 |
| NCU11187 | -1.92 |  | signal sequence receptor alpha subunit |
| NCU02164 | -1.92 |  | hypothetical protein |
| NCU07742 | -1.92 |  | poly(p)/ATP NAD kinase |
| NCU01779 | -1.92 |  | HIRA-interacting protein 5 |
| NCU16572 | -1.92 |  | hypothetical protein |
| NCU09097 | -1.93 |  | hypothetical protein |
| NCU03222 | -1.93 |  | hypothetical protein |
| NCU02358 | -1.94 |  | SDS23 |
| NCU03881 | -1.95 |  | hypothetical protein |
| NCU02270 | -1.95 |  | hypothetical protein |
| NCU00619 | -1.98 |  | hypothetical protein |
| NCU09881 | -1.98 |  | hypothetical protein |
| NCU06382 | -1.98 |  | ABC transporter |
| NCU09251 | -1.99 |  | hypothetical protein |
| NCU01615 | -2.00 |  | hypothetical protein |
| NCU06051 | -2.00 |  | hypothetical protein |
| NCU06207 | -2.00 |  | C-5 sterol desaturase |
| NCU00889 | -2.01 |  | RAB family GTPase |
| NCU07503 | -2.02 |  | hypothetical protein |
| NCU06864 | -2.02 |  | cAMP-independent regulatory protein pac2 |
| NCU03028 | -2.03 |  | deubiquitination-protection protein dph1 |
| NCU05070 | -2.04 |  | hypothetical protein |
| NCU02641 | -2.05 |  | hypothetical protein |
| NCU08621 | -2.05 |  | hypothetical protein |
| NCU09161 | -2.06 |  | translation factor pelota |
| NCU09616 | -2.06 |  | hypothetical protein |
| NCU05386 | -2.07 |  | hypothetical protein |
| NCU00265 | -2.08 |  | hypothetical protein |
| NCU02287 | -2.08 |  | acyl-CoA dehydrogenase-1 |
| NCU03781 | -2.09 |  | CobW domain-containing protein |
| NCU02376 | -2.09 |  | metallo-beta-lactamase domain-containing protein |
| NCU06317 | -2.10 |  | stress response RCI peptide |
| NCU02504 | -2.10 |  | DUF1640 domain-containing protein |
| NCU02767 | -2.10 |  | hyphal anastamosis-6 |
| NCU02157 | -2.12 |  | COQ4 |
| NCU09909 | -2.12 |  | urea active transporter |
| NCU02936 | -2.12 |  | proline oxidase |
| NCU06847 | -2.14 |  | major facilitator superfamily transporter |
| NCU03661 | -2.14 |  | GTPase-activating protein GYP7 |
| NCU04731 | -2.15 |  | short aerial hyphae-2 |
| NCU01419 | -2.16 |  | quinone oxidoreductase |
| NCU01302 | -2.17 |  | hydrolase |
| NCU05561 | -2.17 |  | copper metallothionein |
| NCU06939 | -2.18 |  | endosome-associated ubiquitin isopeptidase |
| NCU03982 | -2.18 |  | glucose regulated protein 78 |
| NCU03137 | -2.19 |  | nuclear elongation and deformation protein 1 |
| NCU03874 | -2.19 |  | hypothetical protein |
| NCU06424 | -2.21 |  | aminomethyl transferase |
| NCU07828 | -2.21 |  | hypothetical protein |
| NCU06218 | -2.22 |  | DUF803 domain membrane protein |
| NCU08167 | -2.24 |  | aerobactin siderophore biosynthesis protein iucB |
| NCU06008 | -2.27 |  | hypothetical protein |
| NCU05986 | -2.29 |  | sucrase/ferredoxin domain-containing protein |
| NCU04637 | -2.32 |  | BAR adaptor protein RVS167 |
| NCU08330 | -2.32 |  | hypothetical protein |
| NCU08707 | -2.34 |  | iron sulfur assembly protein |
| NCU08028 | -2.35 |  | hypothetical protein |
| NCU03254 | -2.37 |  | hypothetical protein |
| NCU00762 | -2.38 |  | glycosylhydrolase family 5-1 |
| NCU00740 | -2.42 |  | hypothetical protein |
| NCU07937 | -2.42 |  | sphingoid long chain base kinase 4 |
| NCU03141 | -2.44 |  | lysophospholipase |
| NCU05970 | -2.45 |  | hypothetical protein |
| NCU16848 | -2.46 |  | hypothetical protein |
| NCU00682 | -2.46 |  | protein kinase A catalytic subunit-2 |
| NCU04443 | -2.46 |  | quinone oxidoreductase |
| NCU08395 | -2.48 |  | pre-mRNA splicing factor Dim1 |
| NCU06052 | -2.48 |  | DnaJ domain-containing protein |
| NCU05512 | -2.48 |  | copper resistance protein Crd2 |
| NCU16397 | -2.49 |  | nuclear polyadenylated RNA-binding protein Nab2 |
| NCU06230 | -2.50 |  | serine/threonine protein kinase-39 |
| NCU05261 | -2.51 |  | ATP-dependent protease La |
| NCU17100 | -2.52 |  | hypothetical protein |
| NCU01060 | -2.55 |  | hypothetical protein |
| NCU03492 | -2.57 |  | inositolphosphorylceramide-B C-26 hydroxylase |
| NCU08270 | -2.57 |  | dicer-like protein 1 |
| NCU03623 | -2.60 |  | ubiquitin-conjugating enzyme E |
| NCU10007 | -2.61 |  | acetate utilization-9 |
| NCU06231 | -2.62 |  | vacuolar amino acid transporter 1 |
| NCU06273 | -2.65 |  | hypothetical protein |
| NCU02665 | -2.70 |  | diacylglycerol O-acyltransferase |
| NCU04142 | -2.72 |  | heat shock protein 80 |
| NCU08292 | -2.73 |  | hypothetical protein |
| NCU07787 | -2.73 |  | clock-controlled gene-14 |
| NCU08994 | -2.76 |  | hypothetical protein |
| NCU10521 | -2.77 |  | glutathione S-transferase-4 |
| NCU09443 | -2.78 |  | hypothetical protein |
| NCU16329 | -2.84 |  | hypothetical protein |
| NCU03802 | -2.87 |  | carnitine biosynthesis-1 |
| NCU00949 | -2.93 |  | hypothetical protein |
| NCU09602 | -2.95 |  | heat shock protein 70-1 |
| NCU02271 | -3.04 |  | YagE family protein |
| NCU02499 | -3.07 |  | DNL zinc finger domain-containing protein |
| NCU03408 | -3.08 |  | hypothetical protein |
| NCU02798 | -3.11 |  | hypothetical protein |
| NCU10028 | -3.12 |  | bax Inhibitor family protein |
| NCU10051 | -3.26 |  | flavohemoglobin |
| NCU08402 | -3.63 |  | zinc-binding alcohol dehydrogenase |
| NCU04697 | -6.75 |  | cyanide hydratase |
| NCU03208 | -7.31 |  | hypothetical protein |

Gene expression values are represented in log_2_ fold change between each of the conditions.

**S1B Table –Genes only modulated in high-Pi**

| **ID** | **low-Pi** | **high-Pi** | **Gene Product Name** |
| --- | --- | --- | --- |
| NCU00337 |  | 3.78 | nuclear export protein Noc3 |
| NCU07748 |  | 3.73 | hypothetical protein |
| NCU07058 |  | 3.60 | small nucleolar ribonucleoprotein complex subunit Utp14 |
| NCU09756 |  | 3.59 | hypothetical protein |
| NCU11175 |  | 3.54 | ATP-dependent RNA helicase drs-1 |
| NCU04439 |  | 3.54 | ATP-dependent RNA helicase dbp-4 |
| NCU07712 |  | 3.53 | ATP-dependent RNA helicase dbp-10 |
| NCU00059 |  | 3.51 | hypothetical protein |
| NCU02497 |  | 3.49 | hypothetical protein |
| NCU04165 |  | 3.48 | low-temperature viability protein ltv1 |
| NCU03321 |  | 3.45 | eukaryotic ribosome biogenesis protein 1 |
| NCU03051 |  | 3.45 | WD domain-containing protein |
| NCU07070 |  | 3.42 | ATP-dependent RNA helicase dbp-9 |
| NCU09349 |  | 3.40 | ATP-dependent RNA helicase has-1 |
| NCU04348 |  | 3.40 | ribosome biogenesis protein |
| NCU02729 |  | 3.37 | transducin family protein |
| NCU02604 |  | 3.36 | hypothetical protein |
| NCU00794 |  | 3.35 | ribosome biogenesis protein Rsa4 |
| NCU02284 |  | 3.32 | nucleolar ATPase Kre33 |
| NCU06485 |  | 3.30 | hypothetical protein |
| NCU08421 |  | 3.29 | hypothetical protein |
| NCU04503 |  | 3.29 | ribosome biogenesis protein Ssf2 |
| NCU03241 |  | 3.28 | FK506-resistant-4 |
| NCU08121 |  | 3.22 | ribosome biogenesis protein |
| NCU09437 |  | 3.21 | hypothetical protein |
| NCU00707 |  | 3.15 | hypothetical protein |
| NCU09380 |  | 3.14 | pumilio domain-containing protein |
| NCU07393 |  | 3.14 | rRNA maturation protein |
| NCU01524 |  | 3.13 | ribosome biogenesis protein BRX1 |
| NCU02880 |  | 3.11 | hypothetical protein |
| NCU00092 |  | 3.08 | rRNA-processing protein efg-1 |
| NCU07011 |  | 3.08 | WD repeat containing protein 36 |
| NCU04485 |  | 3.08 | pre-rRNA processing protein Esf1 |
| NCU04433 |  | 3.07 | cysteine-14 |
| NCU04041 |  | 3.07 | ATP-dependent RNA helicase mak-5 |
| NCU06496 |  | 3.06 | hypothetical protein |
| NCU04196 |  | 3.06 | tRNA (adenine-N(1)-)-methyltransferase catalytic subunit trm61 |
| NCU09131 |  | 3.05 | nucleolar protein 12 |
| NCU01257 |  | 3.04 | hypothetical protein |
| NCU01612 |  | 3.04 | pre-mRNA splicing factor ATP-dependent RNA helicase PRP43 |
| NCU03380 |  | 3.03 | ATP-dependent rRNA helicase spb-4 |
| NCU01115 |  | 3.01 | tRNA-splicing endonuclease subunit SEN2 |
| NCU02236 |  | 2.99 | hypothetical protein |
| NCU07795 |  | 2.97 | DUF1665 domain-containing protein |
| NCU01638 |  | 2.96 | DNA-directed RNA polymerase I subunit RPA1 |
| NCU06520 |  | 2.95 | ATP-dependent RNA helicase dbp-7 |
| NCU07041 |  | 2.94 | ribosomal RNA assembly protein mis3 |
| NCU01259 |  | 2.94 | hypothetical protein |
| NCU02159 |  | 2.93 | hypothetical protein |
| NCU07670 |  | 2.93 | cytochrome-19 |
| NCU05782 |  | 2.92 | ATP-dependent RNA helicase dbp-3 |
| NCU02002 |  | 2.91 | hypothetical protein |
| NCU09528 |  | 2.88 | nucleolar protein 16 |
| NCU09708 |  | 2.88 | PDCD2 C terminal domain-containing protein |
| NCU02879 |  | 2.87 | zinc/iron transporter |
| NCU02511 |  | 2.87 | tRNA (cytosine-5-)-methyltransferase NCL1 |
| NCU03961 |  | 2.86 | WD repeat protein |
| NCU01916 |  | 2.85 | mitochondrial carrier protein RIM2 |
| NCU07871 |  | 2.84 | hypothetical protein |
| NCU05418 |  | 2.84 | deoxyhypusine synthase-1 |
| NCU06217 |  | 2.76 | nucleolar protein 4 |
| NCU01669 |  | 2.76 | putative arginine methyltransferase-3 |
| NCU04047 |  | 2.75 | hypothetical protein |
| NCU05235 |  | 2.75 | ribosome biogenesis protein RLP24 |
| NCU04504 |  | 2.73 | ATP-dependent rRNA helicase rrp-3 |
| NCU08626 |  | 2.71 | photoreactivation-deficient |
| NCU02155 |  | 2.71 | hypothetical protein |
| NCU09304 |  | 2.70 | glutamate-1-semialdehyde 2,1-aminomutase |
| NCU01367 |  | 2.70 | small nucleolar ribonucleoprotein complex subunit |
| NCU16769 |  | 2.67 | flavodoxin and radical SAM domain-containing protein |
| NCU00457 |  | 2.65 | translation initiation factor 4B |
| NCU00310 |  | 2.63 | hypothetical protein |
| NCU00597 |  | 2.63 | deoxyribose-phosphate aldolase 2 |
| NCU08595 |  | 2.60 | ribosome biogenesis protein |
| NCU09488 |  | 2.59 | SDA1 domain-containing protein |
| NCU05894 |  | 2.59 | hypothetical protein |
| NCU03117 |  | 2.58 | inosine-5'-monophosphate dehydrogenase IMD2 |
| NCU00151 |  | 2.57 | hypothetical protein |
| NCU16757 |  | 2.56 | H/ACA ribonucleoprotein complex subunit 1 |
| NCU00552 |  | 2.56 | albino-1 |
| NCU09959 |  | 2.56 | hypothetical protein |
| NCU06943 |  | 2.56 | SIK1 |
| NCU06168 |  | 2.53 | diphthamide biosynthesis protein 2 |
| NCU09870 |  | 2.53 | hypothetical protein |
| NCU01516 |  | 2.53 | mitochondrial co-chaperone GrpE |
| NCU04018 |  | 2.50 | hypothetical protein |
| NCU17240 |  | 2.50 | hypothetical protein |
| NCU04827 |  | 2.49 | hypothetical protein |
| NCU09830 |  | 2.48 | menadione-induced gene-12 |
| NCU01260 |  | 2.47 | hypothetical protein |
| NCU09261 |  | 2.45 | DNA replication complex GINS protein SLD5 |
| NCU04150 |  | 2.45 | ribosomal large subunit biogenesis protein MAK16 |
| NCU05301 |  | 2.44 | methyltransferase |
| NCU01894 |  | 2.43 | hypothetical protein |
| NCU00276 |  | 2.43 | MIP1-like DNA polymerase |
| NCU05251 |  | 2.42 | DNA-directed RNA polymerase I polypeptide |
| NCU04942 |  | 2.42 | methionine permease |
| NCU11219 |  | 2.38 | A/G-specific adenine glycosylase |
| NCU01786 |  | 2.38 | ribose-phosphate pyrophosphokinase II |
| NCU03288 |  | 2.37 | hypothetical protein |
| NCU06336 |  | 2.37 | N2,N2-dimethylguanosine tRNA methyltransferase |
| NCU03560 |  | 2.34 | DNA-directed RNA polymerase III subunit rpc-3 |
| NCU08030 |  | 2.34 | UDP-glucose 4-epimerase Gal10 |
| NCU06214 |  | 2.33 | hypothetical protein |
| NCU07409 |  | 2.30 | hypothetical protein |
| NCU03133 |  | 2.30 | hypothetical protein |
| NCU02256 |  | 2.29 | exosome complex exonuclease Rrp6 |
| NCU01245 |  | 2.27 | hypothetical protein |
| NCU03669 |  | 2.27 | AdoMet-dependent rRNA methyltransferase spb1 |
| NCU00404 |  | 2.26 | RNA processing factor 1 |
| NCU03030 |  | 2.26 | cytochrome-18 |
| NCU00692 |  | 2.26 | chaperone dnaK |
| NCU08310 |  | 2.25 | purine-cytosine permease FCY21 |
| NCU03907 |  | 2.25 | pre-rRNA processing protein |
| NCU02805 |  | 2.25 | diphthamide biosynthesis protein 4 |
| NCU06468 |  | 2.24 | midasin |
| NCU02898 |  | 2.23 | hypothetical protein |
| NCU09958 |  | 2.23 | hypothetical protein |
| NCU09030 |  | 2.23 | hypothetical protein |
| NCU01083 |  | 2.22 | spermidine-2 |
| NCU10185 |  | 2.22 | tRNA isopentenyltransferase |
| NCU07731 |  | 2.20 | hypothetical protein |
| NCU07420 |  | 2.18 | eIF4A |
| NCU07839 |  | 2.17 | ATP-dependent RNA helicase dbp-2 |
| NCU09254 |  | 2.16 | pre-mRNA-splicing factor ATP-dependent RNA helicase PRP16 |
| NCU06306 |  | 2.16 | chromatin remodeling factor 5-1 |
| NCU08287 |  | 2.15 | pyrimidine-3 |
| NCU04159 |  | 2.15 | DUF652 domain-containing protein |
| NCU01785 |  | 2.14 | hypothetical protein |
| NCU07295 |  | 2.13 | translocase of inner mitochondrial membrane 54 |
| NCU06662 |  | 2.11 | mitochondrial carrier protein |
| NCU08510 |  | 2.11 | hypothetical protein |
| NCU16779 |  | 2.07 | hypothetical protein |
| NCU07864 |  | 2.07 | 60S ribosome biogenesis protein Mak11 |
| NCU00355 |  | 2.06 | catalase-3 |
| NCU01993 |  | 2.05 | ethanolaminephosphotransferase |
| NCU01121 |  | 2.04 | hypothetical protein |
| NCU02231 |  | 2.03 | tRNA (guanine-N(1)-)-methyltransferase |
| NCU08441 |  | 1.99 | non-ribosomal peptide synthetase |
| NCU08692 |  | 1.99 | cytochrome a-5 |
| NCU01304 |  | 1.98 | mitochondrial mRNA processing protein PET127 |
| NCU00105 |  | 1.98 | 60S ribosome subunit biogenesis protein NIP7 |
| NCU05551 |  | 1.98 | hypothetical protein |
| NCU06721 |  | 1.95 | peptide chain release factor 1 |
| NCU05287 |  | 1.92 | 50S ribosomal protein L4 |
| NCU06407 |  | 1.92 | vegetative asexual development-3 |
| NCU06469 |  | 1.91 | 54S ribosomal protein L12 |
| NCU05293 |  | 1.90 | rRNA processing protein Rrp8 |
| NCU00768 |  | 1.89 | mRNA binding post-transcriptional regulator |
| NCU05601 |  | 1.84 | cytochrome-2 |
| NCU07541 |  | 1.84 | hypothetical protein |
| NCU16466 |  | 1.80 | nucleic acid-binding protein |
| NCU01855 |  | 1.80 | hypothetical protein |
| NCU04216 |  | 1.79 | adenine-7 |
| NCU01001 |  | 1.79 | hypothetical protein |
| NCU07386 |  | 1.73 | Fe superoxide dismutase |
| NCU09536 |  | -1.52 | hypothetical protein |
| NCU11308 |  | -1.56 | hypothetical protein |
| NCU10721 |  | -1.60 | solute carrier family 35 member B1 protein |
| NCU02777 |  | -1.60 | molybdenum cofactor sulfurase |
| NCU08447 |  | -1.62 | hypothetical protein |
| NCU01382 |  | -1.63 | hypothetical protein |
| NCU06613 |  | -1.65 | ammonium transporter |
| NCU07135 |  | -1.66 | hypothetical protein |
| NCU16725 |  | -1.70 | potassium ion channel Yvc1 |
| NCU09043 |  | -1.71 | caleosin domain-containing protein |
| NCU08978 |  | -1.71 | hypothetical protein |
| NCU05079 |  | -1.77 | MFS peptide transporter |
| NCU08967 |  | -1.80 | arrestin domain-containing protein |
| NCU07545 |  | -1.81 | DUF1183 domain-containing protein |
| NCU00604 |  | -1.82 | hypothetical protein |
| NCU07388 |  | -1.85 | hypothetical protein |
| NCU01289 |  | -1.89 | hypothetical protein |
| NCU07740 |  | -1.91 | hypothetical protein |
| NCU07110 |  | -1.91 | hypothetical protein |
| NCU02146 |  | -1.93 | hypothetical protein |
| NCU00069 |  | -1.93 | hypothetical protein |
| NCU04255 |  | -1.94 | hypothetical protein |
| NCU01759 |  | -1.95 | menadione-induced gene-5 |
| NCU09505 |  | -1.95 | hypothetical protein |
| NCU08273 |  | -1.96 | plasma membrane channel protein |
| NCU11098 |  | -1.97 | UPF0052 domain-containing protein |
| NCU02099 |  | -1.97 | hypothetical protein |
| NCU00627 |  | -1.98 | hypothetical protein |
| NCU01078 |  | -2.00 | D-mandelate dehydrogenase |
| NCU02261 |  | -2.01 | hypothetical protein |
| NCU04538 |  | -2.02 | hypothetical protein |
| NCU01070 |  | -2.02 | hypothetical protein |
| NCU01330 |  | -2.02 | SacI domain-containing protein |
| NCU07080 |  | -2.03 | hypothetical protein |
| NCU08298 |  | -2.04 | cytosolic phospholipase A2 zeta |
| NCU08366 |  | -2.05 | hypothetical protein |
| NCU07661 |  | -2.05 | hypothetical protein |
| NCU09874 |  | -2.06 | hypothetical protein |
| NCU07081 |  | -2.07 | lipase |
| NCU02120 |  | -2.09 | hypothetical protein |
| NCU09848 |  | -2.09 | hypothetical protein |
| NCU09739 |  | -2.10 | all development altered-7 |
| NCU07597 |  | -2.10 | hypothetical protein |
| NCU07134 |  | -2.10 | hypothetical protein |
| NCU09473 |  | -2.11 | 3-ketoacyl-acyl carrier protein reductase |
| NCU06337 |  | -2.12 | hypothetical protein |
| NCU07590 |  | -2.12 | Ser/Thr protein phosphatase |
| NCU08791 |  | -2.12 | catalase-1 |
| NCU09738 |  | -2.14 | alpha-ketoglutarate dependent xanthine dioxygenase |
| NCU00869 |  | -2.14 | hypothetical protein |
| NCU03105 |  | -2.17 | hypothetical protein |
| NCU05915 |  | -2.17 | hypothetical protein |
| NCU11041 |  | -2.17 | hypothetical protein |
| NCU00673 |  | -2.19 | serine protease p2 |
| NCU03240 |  | -2.19 | hypothetical protein |
| NCU03893 |  | -2.21 | short-chain dehydrogenase/reductase SDR |
| NCU08057 |  | -2.22 | short chain dehydrogenase/reductase |
| NCU05309 |  | -2.22 | Cut9 interacting protein Scn1 |
| NCU08155 |  | -2.25 | hypothetical protein |
| NCU07332 |  | -2.26 | hypothetical protein |
| NCU07925 |  | -2.27 | LRP16 |
| NCU04122 |  | -2.27 | malate dehydrogenase |
| NCU11365 |  | -2.27 | aminotransferase |
| NCU05157 |  | -2.28 | cation diffusion facilitator 10 |
| NCU05616 |  | -2.29 | arsenite S-adenosylmethyltransferase |
| NCU00877 |  | -2.30 | hypothetical protein |
| NCU02321 |  | -2.33 | hypothetical protein |
| NCU01862 |  | -2.33 | SWIRM domain-containing protein FUN19 |
| NCU03506 |  | -2.33 | hypothetical protein |
| NCU09692 |  | -2.33 | phosphatidic acid phosphatase beta |
| NCU09513 |  | -2.34 | GTP-binding protein 1 |
| NCU06875 |  | -2.36 | hypothetical protein |
| NCU09085 |  | -2.37 | cyclin |
| NCU10400 |  | -2.38 | phospholipase PldA |
| NCU11216 |  | -2.38 | hypothetical protein |
| NCU04990 |  | -2.40 | fissure |
| NCU12022 |  | -2.41 | hypothetical protein |
| NCU09049 |  | -2.41 | hypothetical protein |
| NCU05257 |  | -2.43 | homeobox and C2H2 transcription fator |
| NCU00268 |  | -2.44 | hypothetical protein |
| NCU09683 |  | -2.45 | hypothetical protein |
| NCU06682 |  | -2.47 | plasma membrane proteolipid 3 |
| NCU00995 |  | -2.48 | hypothetical protein |
| NCU05838 |  | -2.49 | hypothetical protein |
| NCU01830 |  | -2.49 | 4-hydroxyphenylpyruvate dioxygenase |
| NCU09057 |  | -2.49 | hypothetical protein |
| NCU11341 |  | -2.50 | translation initiation factor eIF-2B |
| NCU07607 |  | -2.50 | sugar transporter |
| NCU06265 |  | -2.52 | hypothetical protein |
| NCU05151 |  | -2.53 | phosphoketolase |
| NCU11172 |  | -2.55 | hypothetical protein |
| NCU04669 |  | -2.58 | hypothetical protein |
| NCU17064 |  | -2.60 | 5-nitroimidazole antibiotic resistance protein |
| NCU02596 |  | -2.61 | hypothetical protein |
| NCU09337 |  | -2.62 | Pheromone-regulated membrane protein 1-like |
| NCU00766 |  | -2.64 | hypothetical protein |
| NCU12021 |  | -2.68 | hypothetical protein |
| NCU03654 |  | -2.71 | hypothetical protein |
| NCU01077 |  | -2.71 | hypothetical protein |
| NCU06131 |  | -2.72 | hypothetical protein |
| NCU09724 |  | -2.73 | hypothetical protein |
| NCU01874 |  | -2.73 | hypothetical protein |
| NCU09403 |  | -2.76 | NmrA family protein |
| NCU04006 |  | -2.76 | hypothetical protein |
| NCU01107 |  | -2.76 | short-chain dehydrogenase |
| NCU08425 |  | -2.77 | major facilitator superfamily transporter MFS_1 |
| NCU07349 |  | -2.77 | hypothetical protein |
| NCU00868 |  | -2.81 | hypothetical protein |
| NCU01893 |  | -2.81 | hypothetical protein |
| NCU12034 |  | -2.85 | hypothetical protein |
| NCU04667 |  | -2.87 | hypothetical protein |
| NCU02322 |  | -2.88 | mannose-6-phosphate isomerase |
| NCU02022 |  | -2.89 | hypothetical protein |
| NCU01127 |  | -2.91 | DUF636 domain-containing protein |
| NCU07120 |  | -2.91 | DUF1264 domain-containing protein |
| NCU06890 |  | -2.91 | hypothetical protein |
| NCU00322 |  | -2.92 | hypothetical protein |
| NCU01233 |  | -2.94 | aldose reductase |
| NCU07806 |  | -2.96 | hypothetical protein |
| NCU09629 |  | -3.14 | hypothetical protein |
| NCU09041 |  | -3.15 | L-xylulose reductase |
| NCU09451 |  | -3.18 | hypothetical protein |
| NCU05524 |  | -3.20 | hypothetical protein |
| NCU09627 |  | -3.20 | hypothetical protein |
| NCU02540 |  | -3.21 | meiotic expression up-regulated protein 14 |
| NCU05376 |  | -3.22 | p450 monooxygenase |
| NCU03780 |  | -3.23 | hypothetical protein |
| NCU16318 |  | -3.33 | hypothetical protein |
| NCU04605 |  | -3.39 | hypothetical protein |
| NCU01510 |  | -3.42 | meiotically up-regulated 190 protein |
| NCU01555 |  | -3.51 | hypothetical protein |
| NCU09821 |  | -3.54 | oxidoreductase |
| NCU00247 |  | -3.61 | hypothetical protein |
| NCU10359 |  | -3.62 | neutral ceramidase |
| NCU07337 |  | -3.68 | hypothetical protein |
| NCU03205 |  | -3.79 | hypothetical protein |
| NCU01088 |  | -3.94 | hypothetical protein |
| NCU04148 |  | -3.95 | hypothetical protein |
| NCU07424 |  | -4.02 | hypothetical protein |
| NCU07441 |  | -4.09 | hypothetical protein |
| NCU09603 |  | -4.28 | hypothetical protein |
| NCU09173 |  | -5.34 | hypothetical protein |
| NCU05004 |  | -6.58 | hypothetical protein |

Gene expression values are represented in log_2_ fold change between each of the conditions.

**S1C Table –Genes modulated in both low- and high-Pi**

| **ID** | **low-Pi** | **high-Pi** | **Gene Product Name** |
| --- | --- | --- | --- |
| NCU16992 | 7.40 | 7.33 | mating factor a-1 |
| NCU03921 | 4.55 | -1.59 | mitochondrial chaperone bcs1 |
| NCU02939 | 3.36 | -1.87 | hypothetical protein |
| NCU09489 | 3.27 | 3.88 | phosphoglycerate mutase |
| NCU04528 | 3.05 | -2.04 | laccase precursor |
| NCU09843 | 2.88 | 3.55 | WD repeat containing protein 57 |
| NCU09137 | 2.74 | 3.44 | hypothetical protein |
| NCU03153 | 2.66 | 2.72 | hypothetical protein |
| NCU07149 | 2.54 | -3.04 | hypothetical protein |
| NCU06428 | 2.54 | 2.98 | pseudouridine synthase TruD/Pus7 |
| NCU06227 | 2.47 | 2.37 | hypothetical protein |
| NCU05061 | 2.44 | 3.14 | hypothetical protein |
| NCU04571 | 2.41 | 2.87 | hypothetical protein |
| NCU03430 | 2.37 | -1.77 | hypothetical protein |
| NCU04787 | 2.33 | 4.19 | bfr-2 |
| NCU07752 | 2.26 | -2.80 | hypothetical protein |
| NCU04455 | 2.22 | 3.43 | hypothetical protein |
| NCU01022 | 2.20 | 3.56 | DUF1253 domain-containing protein |
| NCU05252 | 2.17 | 3.64 | deoxyhypusine hydroxylase |
| NCU08281 | 2.14 | -1.59 | hypothetical protein |
| NCU02546 | 2.14 | 3.58 | nucleolar GTP-binding protein 2 |
| NCU00336 | 2.13 | 3.64 | U3 small nucleolar RNA-associated protein 10 |
| NCU16370 | 2.13 | -2.24 | hypothetical protein |
| NCU01503 | 2.13 | 3.36 | pre-rRNA processing protein Tsr1 |
| NCU03794 | 2.12 | 3.59 | periodic tryptophan protein 2 |
| NCU00115 | 2.10 | 3.15 | rRNA-processing protein FCF2 |
| NCU08923 | 2.10 | 2.64 | zinc knuckle domain-containing protein |
| NCU03066 | 2.10 | 3.83 | GTP-binding protein |
| NCU06088 | 2.10 | 3.15 | hypothetical protein |
| NCU07956 | 2.07 | 2.84 | pre-rRNA-processing protein PNO1 |
| NCU09383 | 2.06 | 2.38 | hypothetical protein |
| NCU04647 | 2.05 | 3.04 | actin binding protein |
| NCU07030 | 2.05 | -1.67 | hypothetical protein |
| NCU00727 | 2.03 | 2.82 | hypothetical protein |
| NCU10062 | 2.03 | 2.95 | hypothetical protein |
| NCU03092 | 2.02 | 2.89 | nuclear localization sequence binding protein |
| NCU06409 | 2.02 | 3.55 | ribosome biogenesis protein Kri1 |
| NCU01595 | 2.02 | 3.69 | SOF1 |
| NCU09303 | 2.00 | 3.07 | hypothetical protein |
| NCU07459 | 1.96 | 3.13 | protein arginine N-methyltransferase-1 |
| NCU07547 | 1.95 | 2.74 | 60S acidic ribosomal protein P0 |
| NCU08903 | 1.95 | 3.29 | Nop10 family nucleolar RNA-binding protein |
| NCU06752 | 1.92 | 3.45 | hypothetical protein |
| NCU05650 | 1.92 | 3.48 | karyopherin Kap123 |
| NCU01506 | 1.91 | 2.40 | hypothetical protein |
| NCU03808 | 1.91 | 3.64 | pre-mRNA-splicing factor ATP-dependent RNA helicase PRP16 |
| NCU00777 | 1.89 | 3.53 | methyltransferase |
| NCU04799 | 1.88 | 2.19 | polyadenylate-binding protein |
| NCU11321 | 1.87 | 3.32 | ribosomal L1 domain-containing protein 1 |
| NCU08968 | 1.87 | 2.64 | dimethyladenosine transferase |
| NCU09499 | 1.85 | 3.55 | hypothetical protein |
| NCU05233 | 1.85 | 2.99 | p60 domain-containing protein |
| NCU02237 | 1.84 | 2.32 | GRC3 |
| NCU00501 | 1.83 | 3.46 | nucleolar complex protein 4 |
| NCU05289 | 1.80 | 3.22 | nucleolar GTP-binding protein 1 |
| NCU02066 | 1.79 | 3.21 | ribosome assembly protein Noc2 |
| NCU00038 | 1.79 | 2.65 | C2H2 transcription fator |
| NCU00925 | 1.79 | 3.35 | pescadillo |
| NCU06272 | 1.78 | 3.79 | rRNA biogenesis protein RRP5 |
| NCU01290 | 1.76 | 3.15 | centromere/microtubule-binding protein CBF5 |
| NCU06118 | 1.76 | 4.01 | hypothetical protein |
| NCU03964 | 1.74 | 2.55 | hypothetical protein |
| NCU01809 | 1.74 | 2.66 | AAA family ATPase/60S ribosome export protein Rix7 |
| NCU09521 | 1.71 | 2.45 | ribosome biogenesis protein |
| NCU00301 | 1.71 | 2.67 | Sas10/Utp3 family protein |
| NCU05695 | 1.71 | 3.03 | hypothetical protein |
| NCU04620 | 1.71 | 3.42 | hypothetical protein |
| NCU01675 | 1.70 | 3.37 | ribosomal RNA-processing protein 12 |
| NCU08951 | 1.70 | 3.07 | H/ACA ribonucleoprotein complex subunit 2 |
| NCU00650 | 1.70 | 3.30 | Wdr1p |
| NCU03363 | 1.69 | 2.77 | FRQ-interacting RNA helicase |
| NCU06977 | 1.69 | -2.16 | hypothetical protein |
| NCU00537 | -1.67 | -2.27 | hypothetical protein |
| NCU03840 | -1.70 | -2.30 | hypothetical protein |
| NCU00121 | -1.70 | -2.25 | CLC channel protein |
| NCU08359 | -1.70 | -1.52 | cytidine deaminase |
| NCU04809 | -1.74 | -2.48 | MFS phospholipid transporter |
| NCU08957 | -1.75 | -2.70 | hypothetical protein |
| NCU04604 | -1.77 | -2.85 | hypothetical protein |
| NCU04256 | -1.78 | -2.94 | hypothetical protein |
| NCU01509 | -1.79 | -2.27 | hypothetical protein |
| NCU08738 | -1.80 | -2.45 | MFS peptide transporter |
| NCU05527 | -1.80 | -2.85 | hypothetical protein |
| NCU04493 | -1.83 | -2.57 | hypothetical protein |
| NCU00248 | -1.88 | -3.08 | hypothetical protein |
| NCU03336 | -1.88 | -2.41 | hypothetical protein |
| NCU11395 | -1.89 | -3.18 | S-(hydroxymethyl)glutathione dehydrogenase |
| NCU02704 | -1.90 | -2.18 | branched-chain alpha-keto acid dehydrogenase E2 component |
| NCU06555 | -1.92 | -3.90 | hypothetical protein |
| NCU10603 | -1.92 | -2.70 | hypothetical protein |
| NCU09176 | -1.93 | -3.51 | hypothetical protein |
| NCU08170 | -1.95 | -3.44 | hypothetical protein |
| NCU03913 | -1.96 | -2.38 | 2-oxoisovalerate dehydrogenase beta subunit |
| NCU03634 | -1.97 | -2.71 | hypothetical protein |
| NCU00263 | -1.97 | -1.81 | serin endopeptidase |
| NCU05899 | -1.98 | -3.72 | flotillin domain-containing protein |
| NCU08166 | -1.98 | -3.68 | hypothetical protein |
| NCU09406 | -1.99 | -4.21 | copper amine oxidase |
| NCU06587 | -2.00 | -2.20 | hypothetical protein |
| NCU08561 | -2.03 | -2.59 | succinate/fumarate mitochondrial transporter |
| NCU10987 | -2.05 | -1.80 | MFS transporter Fmp42 |
| NCU01386 | -2.07 | -3.36 | hypothetical protein |
| NCU16742 | -2.08 | -3.48 | hypothetical protein |
| NCU04144 | -2.10 | -2.44 | sterol O-acyltransferase |
| NCU05755 | -2.11 | -4.30 | hypothetical protein |
| NCU09017 | -2.11 | -2.37 | hypothetical protein |
| NCU07233 | -2.11 | -4.52 | hypothetical protein |
| NCU07111 | -2.12 | -2.28 | metallo-beta-lactamase domain-containing protein |
| NCU04266 | -2.14 | -3.61 | hypothetical protein |
| NCU04314 | -2.14 | -3.25 | hypothetical protein |
| NCU00356 | -2.17 | -2.41 | nucleoside transporter |
| NCU06856 | -2.18 | -2.31 | ubiquitin fusion degradation protein |
| NCU05499 | -2.19 | -2.11 | homogentisate 1,2-dioxygenase |
| NCU16738 | -2.22 | -3.85 | hypothetical protein |
| NCU04597 | -2.22 | -2.25 | mitogen-activated protein kinase MAF1 |
| NCU05770 | -2.23 | -2.82 | catalase-2 |
| NCU04112 | -2.24 | -2.96 | hypothetical protein |
| NCU00838 | -2.26 | -3.63 | 3-dehydroshikimate dehydratase |
| NCU03647 | -2.27 | -3.04 | hypothetical protein |
| NCU01816 | -2.31 | -2.11 | allantoicase-l |
| NCU05693 | -2.31 | -2.74 | interferon-induced GTP-binding protein Mx2 |
| NCU07027 | -2.33 | -2.38 | glycogen phosphorylase |
| NCU07495 | -2.34 | -3.33 | sphingolipid long chain base-responsive protein LSP1 |
| NCU05377 | -2.34 | -2.56 | integral membrane protein |
| NCU05828 | -2.34 | -3.64 | hypothetical protein |
| NCU01645 | -2.35 | -2.24 | hypothetical protein |
| NCU00332 | -2.35 | -3.79 | hypothetical protein |
| NCU04847 | -2.37 | -3.12 | cyclin |
| NCU01640 | -2.38 | -2.48 | regulatory particle, non-ATPase-like-4 |
| NCU07915 | -2.45 | -3.10 | integral membrane protein |
| NCU05518 | -2.45 | -2.11 | peroxisomal copper amine oxidase |
| NCU17261 | -2.46 | -2.68 | hypothetical protein |
| NCU09519 | -2.48 | -2.51 | 2,5-diketo-D-gluconic acid reductase A |
| NCU06616 | -2.49 | -3.40 | S-adenosylmethionine-dependent methyltransferase |
| NCU08011 | -2.53 | -2.19 | aminotransferase |
| NCU07108 | -2.53 | -4.86 | hypothetical protein |
| NCU05395 | -2.55 | -2.47 | hypothetical protein |
| NCU09713 | -2.56 | -3.48 | hypothetical protein |
| NCU09520 | -2.56 | -3.44 | hypothetical protein |
| NCU02378 | -2.59 | -2.93 | integral membrane protein |
| NCU08064 | -2.59 | -3.35 | meiotically up-regulated 190 protein |
| NCU06174 | -2.60 | -2.24 | DUF1649 domain-containing protein |
| NCU01080 | -2.60 | -3.53 | glucanase B |
| NCU05435 | -2.64 | -3.02 | hypothetical protein |
| NCU05511 | -2.65 | -3.31 | cytidine/deoxycytidylate deaminase |
| NCU15834 | -2.65 | -2.51 | hypothetical protein |
| NCU06989 | -2.66 | -3.06 | hypothetical protein |
| NCU07979 | -2.66 | -3.40 | hypothetical protein |
| NCU11364 | -2.67 | -4.13 | ketoreductase |
| NCU07318 | -2.68 | -2.63 | mannitol-1-phosphate 5-dehydrogenase |
| NCU05225 | -2.68 | -2.61 | NADH dehydrogenase 64 |
| NCU09559 | -2.69 | -2.46 | clock-controlled gene-9 |
| NCU02502 | -2.69 | -3.78 | S-adenosyl-methionine-sterol-C |
| NCU01487 | -2.71 | -2.42 | hypothetical protein |
| NCU03889 | -2.72 | -2.75 | SNARE |
| NCU04076 | -2.72 | -3.11 | copper resistance-associated P-type ATPase |
| NCU03673 | -2.75 | -3.08 | hypothetical protein |
| NCU09873 | -2.76 | -2.48 | acetate utilization-6 |
| NCU08397 | -2.76 | -5.48 | hypothetical protein |
| NCU05068 | -2.79 | -2.55 | hypothetical protein |
| NCU02483 | -2.82 | -3.55 | hypothetical protein |
| NCU11180 | -2.84 | -2.34 | patatin-like phospholipase domain-containing protein |
| NCU06465 | -2.85 | -2.31 | SUR2 |
| NCU05185 | -2.87 | -2.82 | bifunctional P-450:NADPH-P450 reductase |
| NCU11352 | -2.87 | -2.64 | hypothetical protein |
| NCU09285 | -2.87 | -2.40 | menadione-induced gene-6 |
| NCU00472 | -2.89 | -2.58 | CDC37 |
| NCU08954 | -2.90 | -2.71 | hypothetical protein |
| NCU05653 | -2.90 | -2.60 | carbonic anhydrase |
| NCU11223 | -2.91 | -2.64 | hypothetical protein |
| NCU02613 | -2.92 | -3.91 | hypothetical protein |
| NCU02194 | -2.95 | -2.67 | hypothetical protein |
| NCU09986 | -2.96 | -4.59 | hypothetical protein |
| NCU02718 | -3.03 | -4.27 | hypothetical protein |
| NCU06656 | -3.08 | -2.77 | acetate utilization-15 |
| NCU05582 | -3.12 | -2.65 | ubiquitin fusion degradation protein |
| NCU07578 | -3.17 | -4.29 | peroxisomal adenine nucleotide transporter 1 |
| NCU03735 | -3.18 | -2.40 | hypothetical protein |
| NCU06331 | -3.23 | -2.48 | hypothetical protein |
| NCU10270 | -3.25 | -2.65 | hypothetical protein |
| NCU08694 | -3.34 | -3.08 | hypothetical protein |
| NCU03660 | -3.35 | -5.40 | FK506 suppressor Sfk1 |
| NCU05881 | -3.36 | -2.52 | DUF500 and UBA/TS-N domain-containing protein |
| NCU02463 | -3.36 | -3.48 | bax Inhibitor family protein |
| NCU05985 | -3.37 | -2.82 | glycerol-3-phosphate O-acyltransferase |
| NCU02883 | -3.37 | -2.35 | hypothetical protein |
| NCU08986 | -3.43 | -3.62 | hypothetical protein |
| NCU06585 | -3.45 | -3.40 | Rad4 family protein |
| NCU03847 | -3.45 | -3.45 | hypothetical protein |
| NCU07817 | -3.48 | -4.09 | non-anchored cell wall protein-3 |
| NCU05775 | -3.55 | -3.38 | amino acid transporter |
| NCU05842 | -3.58 | -2.95 | hypothetical protein |
| NCU08171 | -3.61 | -4.95 | anchored cell wall protein-12 |
| NCU06782 | -3.65 | -3.33 | zinc metalloproteinase |
| NCU07082 | -3.66 | -5.03 | aspartyl-tRNA synthetase |
| NCU07853 | -3.69 | -4.48 | uricase |
| NCU06332 | -3.74 | -2.95 | alpha/beta hydrolase |
| NCU09020 | -3.77 | -2.86 | hypothetical protein |
| NCU01546 | -3.81 | -2.61 | coproporphyrinogen III oxidase |
| NCU03016 | -3.86 | -3.94 | hypothetical protein |
| NCU08269 | -3.88 | -3.24 | pyridoxine-4 |
| NCU01891 | -3.90 | -5.20 | hypothetical protein |
| NCU11367 | -3.93 | -4.04 | inositol phospholipid biosynthesis protein Scs3 |
| NCU03714 | -3.98 | -3.73 | thioredoxin |
| NCU08507 | -3.99 | -3.26 | zinc finger protein zpr1 |
| NCU06123 | -4.01 | -3.18 | phosphoketolase |
| NCU09098 | -4.03 | -3.34 | tetracycline transporter |
| NCU02623 | -4.03 | -3.07 | mitochondrial hypoxia responsive domain-containing protein |
| NCU02567 | -4.08 | -4.21 | hypothetical protein |
| NCU00306 | -4.15 | -5.53 | MFS multidrug transporter |
| NCU05338 | -4.16 | -3.58 | hypothetical protein |
| NCU03078 | -4.24 | -2.76 | hypothetical protein |
| NCU04874 | -4.29 | -4.85 | alternative oxidase-3 |
| NCU01066 | -4.34 | -2.65 | l-amino acid oxidase |
| NCU09336 | -4.45 | -3.94 | hypothetical protein |
| NCU09040 | -4.47 | -4.07 | menadione-induced gene-4 |
| NCU04591 | -4.55 | -3.38 | pentachlorophenol monooxygenase |
| NCU02142 | -4.62 | -4.27 | hypothetical protein |
| NCU07569 | -4.78 | -4.44 | hypothetical protein |
| NCU03732 | -4.89 | -4.86 | SIS1 |
| NCU08695 | -5.03 | -4.04 | hypothetical protein |
| NCU04502 | -5.03 | -5.74 | hypothetical protein |
| NCU02630 | -5.11 | -4.42 | heat shock protein 78 |
| NCU01871 | -5.15 | -4.37 | hypothetical protein |
| NCU11338 | -5.27 | -5.40 | hypothetical protein |
| NCU06940 | -5.51 | -4.26 | hypothetical protein |
| NCU07465 | -5.61 | -6.19 | mitochondrial phosphate carrier protein 2 |
| NCU07405 | -5.63 | -5.59 | hypothetical protein |
| NCU01873 | -6.19 | -4.47 | hypothetical protein |
| NCU00104 | -6.37 | -5.94 | heat shock protein 98 |
| NCU09174 | -6.40 | -5.78 | hypothetical protein |
| NCU00701 | -6.70 | -6.98 | lysozyme |
| NCU01754 | -6.86 | -5.71 | alcohol dehydrogenase-1 |
| NCU08720 | -7.19 | -7.36 | hypothetical protein |
| NCU07257 | -7.35 | -7.35 | F-box domain-containing protein |
| NCU02500 | -7.36 | -9.34 | clock-controlled gene-4 |
| NCU16673 | -7.44 | -5.38 | hypothetical protein |
| NCU07351 | -7.69 | -6.87 | alpha-glucuronidase |
| NCU05498 | -8.93 | -8.96 | hypothetical protein |
| NCU00754 | -9.44 | -8.05 | multidrug resistant protein |
| NCU04276 | -9.92 | -8.87 | hypothetical protein |

Gene expression values are represented in log_2_ fold change between each of the conditions.
