# Supplementary material for: mus-52 disruption and metabolic regulation in Neurospora crassa: Transcriptional responses to extracellular phosphate availability
Source: PLoS One. 2018 Apr 18;13(4):e0195871. doi: 10.1371/journal.pone.0195871 (PMC5905970; doi:10.1371/journal.pone.0195871)
Supplement: S2 Table — (DOCX) [file pone.0195871.s002.docx]

**S2 Table. Top 10 most significantly up- or down-regulated differentially expressed genes.**

**.**

| **ID** | **Gene Product Name** | | **GO^(a)^** | **log_2_(Fold change)** |
| --- | --- | --- | --- | --- |
| **S1A Table - FGSC 2489 (high-Pi *vs* low-Pi)** | | | | |
| **Up-regulated genes** | | | | |
| NCU08447 | hypothetical protein | C:integral component of membrane | | 2.6193 |
| NCU06803 | glutamate decarboxylase | F:pyridoxal phosphate binding; P:glutamate metabolic process; F:glutamate decarboxylase activity | | 2.5353 |
| NCU05616 | arsenite S-adenosylmethyltransferase | C:cytoplasm; F:S-adenosylmethionine-dependent methyltransferase activity | | 2.1734 |
| NCU12021 | hypothetical protein | C:integral component of membrane | | 2.1719 |
| NCU03921 | mitochondrial chaperone bcs1 | F:ATP binding; C:integral component of membrane | | 2.1404 |
| NCU09173 | hypothetical protein | C:integral component of membrane | | 2.1182 |
| NCU09604 | hypothetical protein | - | | 2.0864 |
| NCU07752 | hypothetical protein | P:regulation of transcription from RNA polymerase III promoter | | 2.0509 |
| NCU06613 | ammonium transporter | F:ammonium transmembrane transporter; C:integral component of plasma membrane | | 2.0176 |
| NCU12022 | hypothetical protein | C:integral component of membrane | | 1.9821 |
| **Down-regulated genes** | | | | |
| NCU09756 | hypothetical protein | F:ATP-dependent RNA helicase activity | | -2.2450 |
| NCU04697 | cyanide hydratase | F:hydrolase activity, F:cyanide hydratase activity | | -2.2273 |
| NCU02729 | transducin family protein | C:cytosol; P:rRNA processing; C:nucleolus | | -2.1627 |
| NCU06118 | hypothetical protein | P:ribosomal small subunit export from nucleus; C:nucleolus | | -2.1499 |
| NCU06272 | rRNA biogenesis protein RRP5 | F:RNA binding; P:RNA processing; C:nucleolus; C:ribosome | | -2.1379 |
| NCU03321 | eukaryotic ribosome biogenesis protein 1 | C:nucleoplasm; C:preribosome, large subunit precursor | | -2.0729 |
| NCU00337 | nuclear export protein Noc3 | C:nuclear pre-replicative complex; P:DNA replication initiation; C:nucleolus | | -2.0648 |
| NCU04439 | ATP-dependent RNA helicase dbp-4 | C:small-subunit processome; F:ATP binding; F:RNA binding; P:RNA secondary structure unwinding; C:cytosol; P:rRNA processing; C:nucleolus; F:ATP-dependent RNA helicase activity | | -2.0632 |
| NCU07058 | small nucleolar ribonucleoprotein complex subunit Utp14 | C:small-subunit processome; P:endonucleolytic cleavage to generate mature 5'-end of SSU-rRNA from (SSU-rRNA, 5.8S rRNA, LSU-rRNA); P:endonucleolytic cleavage in 5'-ETS of tricistronic rRNA transcript (SSU-rRNA, 5.8S rRNA, LSU-rRNA); C:viral nucleocapsid; P:endonucleolytic cleavage in ITS1 to separate SSU-rRNA from 5.8S rRNA and LSU-rRNA from tricistronic rRNA transcript (SSU-rRNA, 5.8S rRNA, LSU-rRNA); C:nucleolus | | -2.0215 |
| NCU00059 | hypothetical protein | C:Rix1 complex; P:chromatin silencing at centromere; C:chromosome, centromeric region; C:mitotic spindle pole body; C:nuclear heterochromatin | | -2.0208 |
| **S1B Table - FGSC 9568 (high-Pi *vs* low-Pi)** | | | | |
| **Up-regulated genes** | | | | |
| NCU11307 | CCC1 | F:iron ion transmembrane transporter activity; C:integral component of membrane; | | 1.8910 |
| NCU00552 | albino-1 | P:carotenoid biosynthetic process; F:oxidoreductase activity; C:integral component of membrane | | 1.8008 |
| NCU07748 | hypothetical protein | - | | 1.5937 |
| NCU00999 | hypothetical protein | - | | 1.5898 |
| **Down-regulated genes** | | | | |
| NCU09603 | hypothetical protein | - | | -4.0492 |
| NCU03921 | mitochondrial chaperone bcs1 | F:ATP binding; C:integral component of membrane | | -4.0097 |
| NCU07149 | hypothetical protein | P:carbohydrate metabolic process; F:catalytic activity | | -3.9859 |
| NCU02939 | hypothetical protein | - | | -3.7540 |
| NCU04528 | laccase precursor | F:oxidoreductase activity, P:oxidation-reduction process | | -3.6991 |
| NCU04148 | hypothetical protein | C:integral component of membrane | | -3.5691 |
| NCU09627 | hypothetical protein | C:integral component of membrane | | -3.4501 |
| NCU09629 | hypothetical protein | - | | -3.4140 |
| NCU00878 | hypothetical protein | P:transmembrane transport; C:integral component of membrane | | -3.0928 |
| NCU16370 | hypothetical protein | P:ion transmembrane transport; C:integral component of fungal-type vacuolar membrane; F:transporter activity | | -3.0770 |
| **S1C Table - low-Pi (FGSC 9568 *vs* FGSC 2489)** | | | | |
| **Up-regulated genes** | | | | |
| NCU03921 | mitochondrial chaperone bcs1 | F:ATP binding; C:integral component of membrane | | 4.5504 |
| NCU08640 | hypothetical protein | - | | 3.9869 |
| NCU08042 | cellulose degradation regulator-2 | C:nucleus; F:DNA binding; P:regulation of transcription | | 3.4267 |
| NCU00802 | hypothetical protein | C:integral component of membrane | | 3.3940 |
| NCU02939 | hypothetical protein | - | | 3.3598 |
| NCU09489 | phosphoglycerate mutase | C:nucleus; F:phosphatase activity; P:dephosphorylation | | 3.2666 |
| NCU04528 | laccase precursor | F:oxidoreductase activity, oxidoreductase activity | | 3.0501 |
| NCU05001 | cycloheximide-inducible-1 | P:small molecule metabolic process; C:integral component of membrane; F:oxidoreductase activity | | 2.9793 |
| NCU09843 | WD repeat containing protein 57 | C:small-subunit processome; P:maturation of SSU-rRNA from tricistronic rRNA transcript (SSU-rRNA, 5.8S rRNA, LSU-rRNA); F:snoRNA binding; C:mitotic spindle; C:cytosol; C:rDNA heterochromatin; C:t-UTP complex; C:90S preribosome; P:positive regulation of transcription from RNA polymerase I promoter | | 2.8837 |
| NCU04778 | carbonic anhydrase | C:mitochondrial intermembrane space; F:zinc ion binding; P:cellular response to carbon dioxide; P:metabolic process; P:cellular response to oxidative stress; F:carbonate dehydratase activity; P:carbon utilization | | 2.8808 |
| **Down-regulated genes** | | | | |
| NCU04276 | hypothetical protein | - | | -9.9202 |
| NCU00754 | multidrug resistant protein | P:transmembrane transport; C:integral component of membrane | | -9.4387 |
| NCU05498 | hypothetical protein | - | | -8.9348 |
| NCU07351 | alpha-glucuronidase | P:xylan catabolic process | | -7.6860 |
| NCU16673 | hypothetical protein | C:integral component of membrane | | -7.4366 |
| NCU07257 | F-box domain-containing protein | - | | -7.3479 |
| NCU03208 | hypothetical protein | C:integral component of membrane | | -7.3070 |
| NCU08720 | hypothetical protein | - | | -7.1906 |
| NCU01754 | alcohol dehydrogenase-1 | F:zinc ion binding; C:cytoplasm; P:oxidation-reduction process; F:alcohol dehydrogenase (NAD) activity | | -6.8597 |
| NCU04697 | cyanide hydratase | F:hydrolase activity, acting on carbon-nitrogen (but not peptide) bonds; P:nitrogen compound metabolic process; F:cyanide hydratase activity | | -6.7455 |
| **S1D Table - high-Pi (FGSC 9568 *vs* FGSC 2489)** | | | | |
| **Up-regulated genes** | | | | |
| NCU04787 | bfr-2 | C:small-subunit processome; C:nucleolus; C:90S preribosome | | 4.1866 |
| NCU06118 | hypothetical protein | P:ribosomal small subunit export from nucleus; C:nucleolus | | 4.0062 |
| NCU09489 | phosphoglycerate mutase | C:nucleus; F:phosphatase activity; P:dephosphorylation | | 3.8803 |
| NCU03066 | GTP-binding protein | C:nucleolus; C:preribosome, large subunit precursor | | 3.8336 |
| NCU06272 | rRNA biogenesis protein RRP5 | C:small-subunit processome; C:nucleolus; C:ribosome | | 3.7945 |
| NCU00337 | nuclear export protein Noc3 | C:nuclear pre-replicative complex; P:DNA replication initiation; C:Noc2p-Noc3p complex; P:pre-replicative complex assembly involved in nuclear cell cycle DNA replication; F:chromatin binding; P:rRNA processing; C:nucleolus | | 3.7786 |
| NCU07748 | hypothetical protein | - | | 3.7260 |
| NCU01595 | SOF1 | C:small-subunit processome; P:maturation of SSU-rRNA from tricistronic rRNA transcript (SSU-rRNA, 5.8S rRNA, LSU-rRNA); C:mitotic spindle; C:nucleolus; C:Cul4-RING E3 ubiquitin ligase complex | | 3.6947 |
| NCU03808 | pre-mRNA-splicing factor ATP-dependent RNA helicase PRP16 | F:nucleic acid binding; F:ATP binding; C:cytoplasm; P:RNA processing; C:nucleolus; F:ATP-dependent RNA helicase activity | | 3.6440 |
| NCU00336 | U3 small nucleolar RNA-associated protein 10 | C:small-subunit processome; P:endonucleolytic cleavage to generate mature 5'-end of SSU-rRNA from (SSU-rRNA, 5.8S rRNA, LSU-rRNA); F:snoRNA binding; P:endonucleolytic cleavage in 5'-ETS of tricistronic rRNA transcript (SSU-rRNA, 5.8S rRNA, LSU-rRNA); C:mitochondrion; C:rDNA heterochromatin; C:t-UTP complex; P:endonucleolytic cleavage in ITS1 to separate SSU-rRNA from 5.8S rRNA and LSU-rRNA from tricistronic rRNA transcript (SSU-rRNA, 5.8S rRNA, LSU-rRNA); C:90S preribosome; C:mitotic spindle pole body; C:preribosome, small subunit precursor; P:positive regulation of transcription from RNA polymerase I promoter | | 3.6396 |
| **Down-regulated genes** | | | | |
| NCU05498 | hypothetical protein | - | | -8.9633 |
| NCU04276 | hypothetical protein | - | | -8.8745 |
| NCU00754 | multidrug resistant protein | P:transmembrane transport; C:integral component of membrane | | -8.0526 |
| NCU08720 | hypothetical protein | - | | -7.3570 |
| NCU07257 | F-box domain-containing protein | - | | -7.3525 |
| NCU00701 | lysozyme | P:cell wall macromolecule catabolic process; P:peptidoglycan catabolic process | | -6.9823 |
| NCU07351 | alpha-glucuronidase | P:xylan catabolic process; F:alpha-glucuronidase activity | | -6.8706 |
| NCU05004 | hypothetical protein | C:integral component of membrane | | -6.5753 |
| NCU07465 | mitochondrial phosphate carrier protein 2 | F:structural constituent of ribosome; C:mitochondrion; C:integral component of membrane; P:transport; P:translation | | -6.1882 |
| NCU00104 | heat shock protein 98 | C:nuclear envelope; F:ATPase activity; F:ATP binding; P:protein metabolic process; C:cytosol; P:protein refolding; P:protein unfolding; F:misfolded protein binding; P:cellular response to misfolded protein | | -5.9405 |

^a^ Ontologies: C: Cellular Component; P: Biological Process; F: Molecular Function.
