# Supplementary material for: mus-52 disruption and metabolic regulation in Neurospora crassa: Transcriptional responses to extracellular phosphate availability
Source: PLoS One. 2018 Apr 18;13(4):e0195871. doi: 10.1371/journal.pone.0195871 (PMC5905970; doi:10.1371/journal.pone.0195871)
Supplement: S3 Table — (DOCX) [file pone.0195871.s003.docx]

**S3 Table. Log2 fold-change mutagen sensitive gene expression.**

| **ID** | **Gene Product Name** | **FGSC 2489**  **high-Pi *vs* low-Pi** | **FGSC 9568**  **high-Pi *vs* low-Pi** | **low-Pi**  **FGSC 9568 *vs* FGSC 2489** | **high-Pi**  **FGSC 9568 *vs* FGSC 2489** |
| --- | --- | --- | --- | --- | --- |
| NCU09731 | mutagen sensitive-8 | -0.0245 | 0.0335 | -1.5406 | -1.4508 |
| NCU11188 | mutagen sensitive-9 | -0.1958 | -0.0807 | 0.8300 | 0.9451 |
| NCU02379 | mutagen sensitive-10 | 0.3459 | -0.2585 | 0.5470 | -0.0459 |
| NCU04275 | mutagen sensitive-11 | -0.1735 | -0.4933 | 0.9515 | 0.6423 |
| NCU08850 | mutagen sensitive-18 | 0.3545 | 0.4527 | -0.4347 | -0.3179 |
| NCU08598 | quelling-defective-3 (mus-19) | -0.9099 | 0.0631 | 0.6225 | 1.5964 |
| NCU00274 | mutagen sensitive-21 | 0.0638 | -0.3593 | 1.1231 | 0.6983 |
| NCU08730 | mutagen sensitive-23 | -0.5712 | -0.1777 | 1.0358 | 1.4323 |
| NCU02348 | mutagen sensitive-25 | -0.0997 | -0.3512 | 1.0720 | 0.8326 |
| NCU06577 | mutagen sensitive-26 | -0.7226 | -0.2517 | 1.6752 | 2.1421 |
| NCU08484 | mutagen sensitive-27 | -0.3106 | 0.2085 | 0.9290 | 1.4514 |
| NCU07440 | mutagen sensitive-38 | 0.2776 | -0.1197 | -0.1845 | -0.5636 |
| NCU07498 | mutagen sensitive-40 | 0.3888 | -0.8339 | 1.4392 | 0.2338 |
| NCU09516 | mutagen sensitive-41 | -0.5488 | 0.1562 | 0.6438 | 1.3595 |
| NCU02053 | mutagen sensitive-42 | -0.0219 | -0.1824 | -0.0480 | -0.1953 |
| NCU08742 | mutagen sensitive-43 | -0.0254 | 0.0525 | -1.1213 | -1.0149 |
| NCU07066 | mutagen sensitive-44 | 0.1529 | -0.2517 | -0.7355 | -1.1227 |
| NCU04733 | mutagen sensitive-50 | -0.4189 | -0.0931 | 1.3316 | 1.6639 |
| NCU08290 | mutagen sensitive-51 | -0.0260 | -0.1960 | 0.5632 | 0.4032 |
| NCU00077 | mutagen sensitive-52 | 0.7645 | -0.1041 | -0.1698 | -1.0346 |
| NCU06264 | mutagen sensitive-53 | 1.2975 | -1.4756 | 2.3019 | -0.4646 |
| NCU08346 | unknown-1 (mus-58) | -0.6918 | 0.1938 | -1.6607 | -0.7430 |
| NCU02751 | mutagen sensitive-59 | -0.5069 | 0.0532 | -0.0842 | 0.4951 |
| NCU07457 | crossover junction endonuclease mus-81 | -0.3096 | 0.2091 | -0.8456 | -0.3092 |

highlighted in red (p-value < 0.05)
