# Supplementary material for: mus-52 disruption and metabolic regulation in Neurospora crassa: Transcriptional responses to extracellular phosphate availability
Source: PLoS One. 2018 Apr 18;13(4):e0195871. doi: 10.1371/journal.pone.0195871 (PMC5905970; doi:10.1371/journal.pone.0195871)
Supplement: S4 Table — (DOCX) [file pone.0195871.s004.docx]

**S4 Table. Log2 fold-change Kinase gene expression.**

| **ID** | **Gene Product Name** | **low-Pi**  **FGSC 9568 *vs* FGSC 2489** | **high-Pi**  **FGSC 9568 *vs* FGSC 2489** |
| --- | --- | --- | --- |
| NCU00587 | Osmotic sensitive-5 | 1.5895 | -0.2607 |
| NCU00682 | Protein kinase A catalytic subunit-2 | -2.4630 | -1.9417 |
| NCU00685 | Casein kinase I isoform delta | -2.9361 | -2.7126 |
| NCU00914 | Serine/threonine protein kinase-16 | -1.8947 | -0.8330 |
| NCU01500 | Nicotinamide riboside kinase 1 | -1.2707 | -1.9120 |
| NCU01728 | 6-phosphofructo-2-kinase | -2.2238 | -3.0403 |
| NCU02237 | GRC3 | 1.8395 | 2.3230 |
| NCU02712 | Acetate kinase | 0.1741 | -1.6035 |
| NCU02776 | Cell division control protein Cdc6 | 1.8706 | 1.9057 |
| NCU03796 | Serine/threonine protein kinase-24 | -1.7051 | -0.9661 |
| NCU03894 | Serine/threonine protein kinase-4 | -4.6422 | -1.0584 |
| NCU03970 | Riboflavin kinase | -1.7979 | -1.6491 |
| NCU04005 | Casein kinase-1b | -1.7932 | -0.8465 |
| NCU05658 | Serine/threonine protein kinase-36 | -0.3751 | -1.9050 |
| NCU06005 | Glycerol kinase | -1.3875 | -1.9581 |
| NCU06230 | Serine/threonine protein kinase-39 | -2.4965 | -2.2803 |
| NCU06422 | Serine/threonine protein kinase-42 | 0.9420 | 1.9351 |
| NCU06638 | Serine/threonine protein kinase-46 | 2.1863 | 0.8668 |
| NCU07626 | Thermoresistant gluconokinase | -2.2174 | -4.4861 |
| NCU07722 | Ribosome biogenesis-40 | 0.1139 | 1.5350 |
| NCU07742 | Poly(p)/ATP NAD kinase | -1.9212 | -1.0718 |
| NCU07914 | Phosphoglycerate kinase | -1.5383 | -0.6454 |
| NCU09212 | Calcium/calmodulin-dependent kinase-4 | -2.4225 | -3.6809 |
| NCU10351 | Pyridoxine kinase | -1.4154 | -1.8179 |
| NCU11353 | D-xylulose kinase | -1.7829 | -1.2685 |
| NCU11410 | Cell division cycle 7-like | 1.0161 | 1.6786 |
| NCU16698 | Twinfilin-1 | -0.8851 | -1.6116 |
