# Supplementary material for: mus-52 disruption and metabolic regulation in Neurospora crassa: Transcriptional responses to extracellular phosphate availability
Source: PLoS One. 2018 Apr 18;13(4):e0195871. doi: 10.1371/journal.pone.0195871 (PMC5905970; doi:10.1371/journal.pone.0195871)
Supplement: S5 Table — (DOCX) [file pone.0195871.s005.docx]

**S5 Table. Log2 fold-change circadian clock-related gene expression.**

| **ID** | **Gene Product Name** | **low-Pi**  **FGSC 9568 *vs* FGSC 2489** | **high-Pi**  **FGSC 9568 *vs* FGSC 2489** |
| --- | --- | --- | --- |
| NCU00489 | cytoplasmic ribosomal protein-10 | 0.475 | 0.934 |
| NCU00701 | Lysozyme | -6.700 | -6.982 |
| NCU00716 | non-anchored cell wall protein-5 | -2.825 | -1.286 |
| NCU00902 | white collar-2 | 0.643 | 0.554 |
| NCU01418 | clock-controlled gene-6 | -0.540 | 0.002 |
| NCU01452 | 40S ribosomal protein S1 | 0.409 | 0.678 |
| NCU01555 | hypothetical protein | -1.536 | -3.508 |
| NCU02265 | frequency – frq | 0.478 | 0.934 |
| NCU02289 | ubiquitin-conjugating enzyme E2 | -0.826 | -0.846 |
| NCU02356 | white collar-1 | -2.516 | -1.739 |
| NCU02455 | FKBP-type peptidyl-prolyl cis-trans isomerase | -0.007 | -0.283 |
| NCU02596 | hypothetical protein | 0.079 | -2.614 |
| NCU03038 | 40S ribosomal protein S13 | 0.379 | 0.959 |
| NCU03363 | FRQ-interacting RNA helicase | 1.692 | 2.768 |
| NCU05495 | clock-controlled gene-16 | -0.618 | -0.698 |
| NCU06870 | serine palmitoyl CoA transferase subunit LcbA | -0.630 | 0.032 |
| NCU06895 | hypothetical protein | -0.532 | -0.940 |
| NCU06977 | hypothetical protein | 1.689 | -2.156 |
| NCU07290 | nuclear localization protein | 0.337 | 0.485 |
| NCU07569 | hypothetical protein | -4.779 | -4.440 |
| NCU07787 | clock-controlled gene-14 - ccg-14 | -2.729 | -1.796 |
| NCU08457 | easily wettable - ccg-2 | 0.431 | 0.414 |
| NCU08907 | clock-controlled gene-13 - ccg-13 | -3.619 | -1.965 |
| NCU08936 | clock-controlled gene-15 | -0.975 | -0.439 |
| NCU08949 | hypothetical protein | -4.170 | -2.959 |
| NCU09559 | clock-controlled gene-9 | -2.688 | -2.460 |
| NCU09686 | clock-controlled gene-8 | 0.020 | -0.642 |
| NCU09864 | 2-oxoisovalerate dehydrogenase alpha subunit | -0.850 | -1.016 |
