# Supplementary material for: mus-52 disruption and metabolic regulation in Neurospora crassa: Transcriptional responses to extracellular phosphate availability
Source: PLoS One. 2018 Apr 18;13(4):e0195871. doi: 10.1371/journal.pone.0195871 (PMC5905970; doi:10.1371/journal.pone.0195871)
Supplement: S6 Table — (DOCX) [file pone.0195871.s006.docx]

**S6 Table. Comparison of the gene expression levels assayed by RNA-Seq and qPCR approaches.**

| **ID** | **Gene Product Name** | **low-Pi**  **FGSC 9568 *vs* FGSC 2489** | |  | **high-Pi**  **FGSC 9568 *vs* FGSC 2489** | |
| --- | --- | --- | --- | --- | --- | --- |
|  |  | **RNA-seq** | **qPCR** |  | **RNA-seq** | **qPCR** |
| NCU00017 | hypothetical protein | 1.92 | 3.10 |  | 0.50 | 0.34 |
| NCU00038 | C2H2 transcription factor | 1.79 | 1.86 |  | 2.65 | 3.42 |
| NCU00306 | MFS multidrug transporter | -4.15 | -4.16 |  | -5.53 | -5.03 |
| NCU01386 | hypothetical protein | -2.07 | -2.64 |  | -3.36 | -3.64 |
| NCU02142 | hypothetical protein | -4.62 | -4.22 |  | -4.27 | -4.57 |
| NCU02499 | DNL zinc finger domain-containing protein | -3.07 | -3.28 |  | -2.01 | -1.20 |
| NCU03643 | cutinase transcription factor 1 beta | -1.76 | -0.74 |  | -1.69 | 0.59 |
| NCU03649 | hypothetical protein | 2.18 | 3.08 |  | 0.28 | -0.18 |
| NCU04597 | mitogen-activated protein kinase MAF1 | -2.22 | -2.72 |  | -2.25 | -0.63 |
| NCU05257 | homeobox and C2H2 transcription factor | -1.47 | -1.20 |  | -2.43 | -2.51 |
| NCU06977 | hypothetical protein | 1.69 | 2.08 |  | -2.16 | -1.85 |
| NCU08042 | cellulose degradation regulator-2 | 3.43 | 3.70 |  | -0.54 | -0.07 |
| NCU08507 | zinc finger protein zpr1 | -3.99 | -2.50 |  | -3.26 | -3.28 |
