# Supplementary material for: mus-52 disruption and metabolic regulation in Neurospora crassa: Transcriptional responses to extracellular phosphate availability
Source: PLoS One. 2018 Apr 18;13(4):e0195871. doi: 10.1371/journal.pone.0195871 (PMC5905970; doi:10.1371/journal.pone.0195871)
Supplement: S7 Table — (DOCX) [file pone.0195871.s007.docx]

**S7 Table. Genes potentially involved in the mating-type mechanism.**

| **ID** | **Gene Product Name** |
| --- | --- |
| NCU00022 | hypothetical protein |
| NCU00102 | hypothetical protein |
| NCU00136 | mitochondrial translation optimization protein |
| NCU00164 | chromatin remodelling factor 4-2 |
| NCU00180 | hypothetical protein |
| NCU00250 | hypothetical protein |
| NCU00281 | UDP-glucose,sterol transferase |
| NCU00714 | heat shock protein STI1 |
| NCU00724 | hypothetical protein |
| NCU00735 | hypothetical protein |
| NCU00741 | gliotoxin biosynthesis protein GliK |
| NCU00774 | hypothetical protein |
| NCU00919 | ATP-dependent RNA helicase rok-1 |
| NCU01041 | mitochondrial metalloendopeptidase OMA1 |
| NCU01144 | hypothetical protein |
| NCU01301 | 60S ribosomal subunit assembly/export protein loc-1 |
| NCU01329 | hypothetical protein |
| NCU01331 | snRNP and snoRNP protein |
| NCU01427 | albino-3 |
| NCU01458 | hypothetical protein |
| NCU01502 | small nucleolar ribonucleoprotein complex subunit |
| NCU01545 | autophagy protein 8 |
| NCU01561 | hypothetical protein |
| NCU01651 | hypothetical protein |
| NCU01748 | hypothetical protein |
| NCU01769 | hypothetical protein |
| NCU01792 | heat shock protein 90a |
| NCU01835 | hypothetical protein |
| NCU01985 | cysteine-11 |
| NCU01997 | ABC transporter |
| NCU02061 | hypothetical protein |
| NCU02074 | endoplasmic oxidoreductin-1 |
| NCU02100 | LMBR1 domain-containing protein 1 |
| NCU02106 | hypothetical protein |
| NCU02118 | palmitoyltransferase PFA4 |
| NCU02138 | hypothetical protein |
| NCU02197 | riboflavin aldehyde-forming enzyme |
| NCU02397 | isoleucine-valine-5 |
| NCU02494 | hypothetical protein |
| (*) NCU02500 | clock-controlled gene-4 |
| NCU02523 | hypothetical protein |
| NCU02528 | hypothetical protein |
| NCU02533 | DNA-directed RNA polymerase I and III polypeptide |
| NCU02611 | multiple RNA-binding domain-containing protein 1 |
| NCU02612 | hypothetical protein |
| NCU02640 | hypothetical protein |
| NCU02659 | hypothetical protein |
| NCU02765 | RNA binding protein |
| NCU02910 | hypothetical protein |
| NCU03018 | small nucleolar ribonucleoprotein complex subunit |
| NCU03022 | F-box domain-containing protein |
| NCU03023 | phenol 2-monooxygenase |
| NCU03131 | FAD dependent oxidoreductase superfamily |
| NCU03188 | sugar 1,4-lactone oxidase |
| NCU03365 | hypothetical protein |
| NCU03396 | nucleolar protein nop-58 |
| NCU03416 | telomeric repeat binding factor 1 |
| NCU03529 | hypothetical protein |
| NCU03628 | polarity defective-5 |
| NCU03659 | protein kinase-3 |
| NCU03686 | tall aerial hyphae-3 |
| NCU03702 | rRNA 2'-O-methyltransferase fibrillarin |
| NCU03715 | kinesin-3 |
| NCU03737 | elongation factor Tu |
| NCU03753 | clock-controlled gene-1 |
| NCU03797 | ubiquitin C-terminal hydrolase |
| NCU03817 | FMI1 protein |
| NCU03853 | peptidyl-prolyl cis-trans isomerase |
| NCU03952 | U3 small nucleolar ribonucleoprotein Mpp10 |
| NCU03991 | sterol O-acyltransferase 1 |
| NCU04013 | yellow-1 |
| NCU04016 | phosphoglycerate mutase |
| NCU04038 | hypothetical protein |
| NCU04153 | pseudouridine synthase |
| NCU04169 | hypothetical protein |
| NCU04172 | hsp70-like protein |
| NCU04221 | trehalase-2 |
| NCU04268 | hypothetical protein |
| NCU04273 | RNA-binding protein |
| NCU04452 | menadione-induced gene-3 |
| NCU04523 | hypothetical protein |
| NCU04543 | hypothetical protein |
| NCU04583 | acetyltransferase |
| NCU04618 | hypothetical protein |
| NCU04635 | hypothetical protein |
| NCU04695 | methyltransferase-UbiE family protein |
| NCU04765 | hypothetical protein |
| NCU04855 | C2HC5 finger protein |
| NCU04872 | hypothetical protein |
| NCU04897 | hypothetical protein |
| NCU05049 | hypothetical protein |
| NCU05141 | hypothetical protein |
| NCU05160 | ATP-dependent Zn protease |
| NCU05222 | CTLH domain-containing protein |
| NCU05264 | pyrimidine 5'-nucleotidase |
| NCU05387 | hydrolase |
| NCU05488 | RNA-binding protein Vip1 |
| NCU05534 | hypothetical protein |
| NCU05537 | fumarylacetoacetase |
| NCU05563 | hypothetical protein |
| NCU05600 | serine/threonine protein kinase-33 |
| NCU05692 | hypothetical protein |
| NCU05780 | glutathione S-transferase-1 |
| NCU05850 | rubredoxin-NAD(+) reductase |
| NCU05900 | hypothetical protein |
| NCU06043 | GPR/FUN34 family protein |
| NCU06090 | hypothetical protein |
| NCU06261 | uracil phosphoribosyltransferase |
| NCU06290 | hypothetical protein |
| NCU06387 | hypothetical protein |
| NCU06425 | hypothetical protein |
| NCU06443 | hypothetical protein |
| NCU06506 | hypothetical protein |
| NCU06524 | protease inhibitor |
| NCU06553 | hypothetical protein |
| NCU06577 | mutagen sensitive-26 |
| NCU06586 | AN1 zinc finger protein |
| NCU06597 | hypothetical protein |
| NCU06651 | AN1-type zinc finger protein |
| NCU06761 | sphingosine-1-phosphate lyase |
| NCU06780 | tRNA (uracil-5-)-methyltransferase |
| NCU06845 | short chain dehydrogenase/reductase |
| NCU06860 | MFS multidrug transporter |
| NCU06969 | phosphoinositide 3-phosphate phosphatase |
| NCU07037 | hypothetical protein |
| NCU07046 | hypothetical protein |
| NCU07063 | hypothetical protein |
| NCU07098 | hypothetical protein |
| NCU07166 | hypothetical protein |
| NCU07178 | hypothetical protein |
| NCU07235 | hypothetical protein |
| NCU07273 | hypothetical protein |
| NCU07287 | hypothetical protein |
| NCU07309 | guanine deaminase |
| NCU07311 | hypothetical protein |
| NCU07331 | U3 small nucleolar ribonucleoprotein Lcp5 |
| NCU07434 | short-chain dehydrogenase/reductase SDR |
| NCU07439 | hypothetical protein |
| NCU07452 | menadione-induced gene-2 |
| NCU07656 | hypothetical protein |
| NCU07687 | hypothetical protein |
| NCU07741 | 1-acyl-sn-glycerol-3-phosphate acyltransferase 2 |
| NCU07769 | hypothetical protein |
| NCU07851 | superoxide dismutase 1 copper chaperone |
| NCU07888 | hypothetical protein |
| NCU07916 | phenol 2-monooxygenase |
| NCU07921 | hypothetical protein |
| NCU07942 | hypothetical protein |
| NCU08056 | ABC drug exporter AtrF |
| NCU08173 | early conidial development-2 |
| NCU08295 | RNA-binding La domain-containing protein |
| NCU08323 | bystin |
| NCU08372 | triacylglycerol lipase |
| NCU08407 | MFS transporter |
| NCU08418 | tripeptidyl-peptidase |
| NCU08439 | leptomycin B resistance protein pmd1 |
| NCU08476 | hypothetical protein |
| NCU08478 | hypothetical protein |
| NCU08518 | hypothetical protein |
| NCU08549 | UDP-galactose 4-epimerase |
| NCU08616 | unknown-18 |
| NCU08641 | hypothetical protein |
| NCU08663 | nonsense-mediated mRNA decay protein 3 |
| NCU08677 | hypothetical protein |
| NCU08767 | serine/threonine protein kinase-52 |
| NCU08769 | conidiation-6 |
| NCU08895 | PNS1 |
| NCU08900 | FMN binding oxidoreductase |
| NCU08904 | 20S-pre-rRNA D-site endonuclease NOB1 |
| NCU09210 | dyp-type peroxidase |
| NCU09263 | anchored cell wall protein-4 |
| NCU09364 | heat shock protein 30 |
| NCU09482 | hypothetical protein |
| NCU09534 | peroxiredoxin HYR1 |
| NCU09535 | hypothetical protein |
| NCU09541 | hypothetical protein |
| NCU09571 | tRNA (adenine-N(1)-)-methyltransferase non-catalytic subunit trm6 |
| NCU09579 | retinol dehydrogenase 12 |
| NCU09613 | hypothetical protein |
| NCU09674 | O-methyltransferase family 3 |
| NCU09676 | hypothetical protein |
| NCU09799 | hypothetical protein |
| NCU10034 | retrograde vesicle-mediated transporter Get1 |
| (*) NCU16992 | mating factor a-1 |

(*) Removed manually
