# Supplementary material for: mus-52 disruption and metabolic regulation in Neurospora crassa: Transcriptional responses to extracellular phosphate availability
Source: PLoS One. 2018 Apr 18;13(4):e0195871. doi: 10.1371/journal.pone.0195871 (PMC5905970; doi:10.1371/journal.pone.0195871)
Supplement: S8 Table — (DOCX) [file pone.0195871.s008.docx]

**S8 Table. *In silico* prediction of the DNA-binding motif for the regulator MAT1-1-1 (5’-CTATTGAG-3’).**

| **D** | **Gene Product Name** | **Consensus sequence (position)** |
| --- | --- | --- |
| NCU00039 | hypothetical protein | 706 |
| NCU00068 | hypothetical protein | 829 |
| NCU00100 | hypothetical protein | 684 |
| NCU00101 | pbn-1 | 811 |
| NCU00159 | Sin3-associated polypeptide Sap18 | 315 |
| NCU00720 | tricarboxylic acid-17 | 264 |
| NCU00886 | hypothetical protein | 616 |
| NCU01014 | acyltransferase | 888 |
| NCU01230 | serum paraoxonase/arylesterase | 702 |
| NCU01443 | seryl-tRNA synthetase | 340 |
| NCU01470 | vacuolar protein 8 | 588 |
| NCU01734 | hypothetical protein | 881 |
| NCU01750 | hypothetical protein | 487 |
| NCU02081 | hypothetical protein | 133 |
| NCU03049 | flavin-binding monooxygenase | 201 |
| NCU03073 | hypothetical protein | 666 |
| NCU03575 | isoleucyl-tRNA synthetase | 810 |
| NCU03576 | hym1-like | 597 |
| NCU03579 | alanine racemase | 160 |
| NCU03644 | hypothetical protein | 842 |
| NCU03645 | hypothetical protein | 15 |
| NCU03699 | zinc finger containing protein | 131 |
| NCU03864 | hypothetical protein | 94 |
| NCU04033 | covalently-linked cell wall protein | 33 |
| NCU04059 | hypothetical protein | 27 |
| NCU04134 | hypothetical protein | 103 |
| NCU04689 | hypothetical protein | 513 |
| NCU04922 | hypothetical protein | 547 |
| **(*) NCU05038** | **2OG-Fe(II) oxygenase** | **986** |
| NCU05228 | hypothetical protein | 289 |
| NCU05521 | pathogenicity protein | 562 |
| NCU05754 | hypothetical protein | 771 |
| NCU05787 | hypothetical protein | 726 |
| NCU06320 | hypothetical protein | 547 |
| NCU06507 | hypothetical protein | 421 |
| NCU06538 | hypothetical protein | 24 |
| NCU06539 | hypothetical protein | 609 |
| NCU06653 | hypothetical protein | 888 |
| NCU07227 | hypothetical protein | 303 |
| NCU07462 | hypothetical protein | 494 |
| NCU07726 | bcp-1 | 317 |
| NCU07749 | hypothetical protein | 892 |
| NCU08162 | arginine-10 | 354 |
| NCU08524 | hypothetical protein | 842 |
| NCU08575 | hypothetical protein | 88 |
| NCU08812 | hypothetical protein | 833 |
| NCU08813 | hypothetical protein | 508 |
| NCU08822 | hypothetical protein | 444 |
| NCU08964 | 60S ribosomal protein L10 | 56 |
| NCU09080 | hypothetical protein | 601 |
| NCU09227 | kelch repeat-containing protein | 763 |
| NCU09256 | hypothetical protein | 667 |
| NCU09267 | copper radical oxidase | 317 |
| NCU09486 | glycosyl hydrolase family 13-3 | 752 |
| NCU09568 | hypothetical protein | 905 |
| NCU09594 | seryl-tRNA synthetase | 693 |
| NCU09714 | hypothetical protein | 483 |
| NCU09768 | hypothetical protein | 117 |
| NCU09831 | hypothetical protein | 456 |
| NCU11357 | cell cycle control protein | 753 |
| NCU16303 | hypothetical protein | 338 |
| NCU16361 | hypothetical protein | 855 |
| NCU16473 | hypothetical protein | 463 |
| NCU16550 | hypothetical protein | 513 |
| NCU16680 | hypothetical protein | 558 |

(*) Gene subtracted from the differential expression analysis as the result of the location of the consensus sequence in its promoter region.
